# Beyond a single species: Mapping virulence traits across the redefined *Fusobacterium nucleatum* complex

**DOI:** 10.1080/21505594.2026.2629110

**Published:** 2026-02-08

**Authors:** Marietta Wolf, Konstantin J. Scholz, Ali Al-Ahmad, Thorsten Steinberg, Anne Kruse, Sama Rezasoltani, Fabian Cieplik, Georg Conrads

**Affiliations:** a Department of Operative Dentistry and Periodontology, Center of Dental Medicine, Medical Center & Medical Faculty, University of Freiburg, Freiburg i. Br., Germany; b Department of Oral Biotechnology, Center of Dental Medicine, Medical Center & Medical Faculty, University of Freiburg, Freiburg i Br., Germany; c Division of Oral Microbiology and Immunology, Department of Operative Dentistry, Periodontology and Preventive Dentistry, Rheinisch-Westfälische Technische Hochschule (RWTH) University Hospital, Aachen, Germany

**Keywords:** *Fusobacterium nucleatum*, *Fusobacterium animalis*, *Fusobacterium vincentii*, *Fusobacterium polymorphum*, *Fusobacterium watanabei*, *Fusobacterium* taxonomy and virulence factors

## Abstract

*Fusobacterium nucleatum* was long regarded as a single species and later subdivided into four subspecies (*nucleatum, polymorphum, animalis, vincentii/fusiforme*). In 2022, these subspecies were validated as separate species and further members of the *F. nucleatum* complex have been proposed (*F. watanabei, F. paranimalis* sp. nov.). Given the increasing evidence linking *F. nucleatum* to various diseases, identifying (sub-)species-specific virulence factors has become essential. Infection in mammalian hosts depends on virulence factors that can be surface-exposed, released into the extracellular environment, or injected directly into host cells. This narrative review aims to address the different pathogenic potentials of each former subspecies. These differences range from adhesin diversity and metabolic adaptations, the repertoire of ABC transporters, lyases and type IV conjugative pili to the capability to invade tissues, evade the immune system and form biofilms and outer membrane vesicles.

## Graphical abstract

**Figure:**
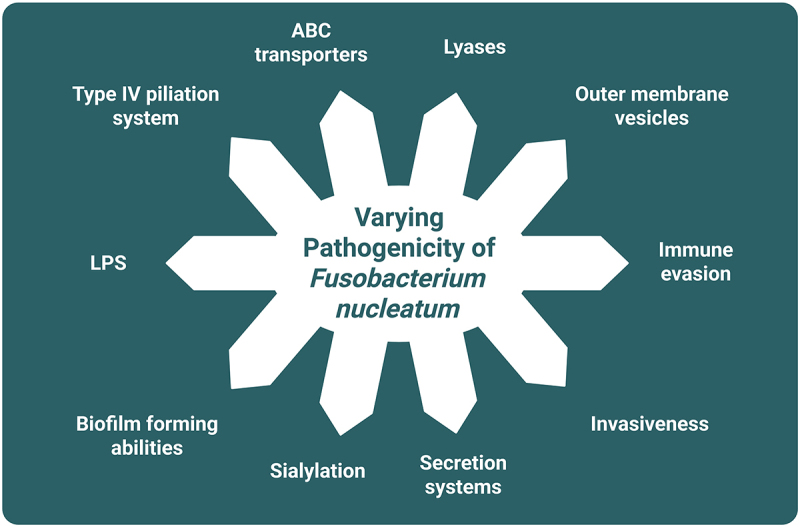

## Introduction

*Fusobacterium nucleatum sensu lato (Fn)* is frequently found in the human oral cavity and has been increasingly recognized for its role in various extraoral infections and systemic diseases like periodontitis, several malignancies, atherosclerosis, diabetes or adverse pregnancy outcome [1–8]. It is a Gram-negative, non-motile, obligate anaerobic rod bacterium.

Historically regarded as a single species, *Fn* has undergone significant taxonomic revision. Former subspecies— *Fusobacterium nucleatum* subspecies *nucleatum*, subspecies *polymorphum*, subspecies *vincentii* (also referred to as *fusiforme*), and subspecies *animalis*—have now been recognized as distinct species: *F. nucleatum* (*sensu stricto*; *Fnuc*), *F. animalis* (*Fan*), *F. polymorphum* (*Fpo*), and *F. vincentii* (*Fvn*), highlighting the genetic and phenotypic diversity within this group [9] (abbreviations have previously been suggested by Zepeda-Rivera et al. [10]). Further species belonging to this *Fn* complex have been suggested: *F. watanabei* (*Fwat*) and *F. paranimalis* sp. nov [11]. These species exhibit distinct ecological preferences and capacities for horizontal gene transfer (HGT), with *Fpo* demonstrating a notably higher propensity for HGT, which complicates strain-level characterization [12,13]. Such diversity underscores the importance of precise species and strain-level identification to better understand their pathogenic potential, as recently reviewed by our group [14]. Genomic comparisons show considerable variation in genome size across former subspecies: *Fpo* and *Fan* possess the largest genomes (~2.6 and ~2.5 Mb), whereas *Fvn* has the smallest (~2.2 Mb) [15].

*Fn*’s immunogenic potential relies on virulence factors (VFs), which vary between former subspecies and can be surface-expressed, secreted, or injected into host cells. These include type V secretion systems, immune modulation, and nutrient acquisition mechanisms [15,16]. The ability of *Fn* to modulate the host’s immune system is a key factor in its persistence and virulence within the host environment. While certain VFs are linked to specific species, their presence is not exclusive, and some strains may lack them. VF gene presence also doesn’t guarantee expression or functionality. Limited strain representation in datasets can skew results, making rare VFs appear absent. Furthermore, inconsistent (sub-)species classification methods reduce the reliability of their associations with virulence profiles, thus, such correlations should be interpreted with caution. Furthermore, despite thorough research and careful evaluation of the literature, it cannot be completely ruled out that the *Fn* species classification used in the cited publications may not reflect taxonomically secured identifications, particularly when methods for species determination are not explicitly stated. These limitations weaken the reliability of any definitive associations between former subspecies and virulence profiles and need to be considered when reading this review.

For simplicity, this review refers to the former *Fn* species complex collectively as *Fn*, whereas the individual species are abbreviated according to the nomenclature proposed by Zepeda-Rivera et al. as *Fnuc, Fan, Fpo, Fvn* [10], and *Fwat*. Although some of the cited publications use the term *F. animalis* C1 instead of *Fwat*, we consistently refer to this taxon as *Fwat* to clearly distinguish it from *Fan*, which has previously also been described as *F. animalis* Clade 2 (C2) [17].

This review aims to summarize the current knowledge on the virulence factors of different *Fn* species, emphasizing the importance of (sub-)species-specific differences in pathogenic potential and the implications for diagnosis, treatment, and understanding of *Fusobacterium*-associated diseases.

## Taxonomic controversies

The taxonomy of the *Fn* complex is still subject to extensive debate. While the survey from the classical four subspecies to species has been described recently [14,18], controveries regarding further species go on. Generally, confusion in the literature partly arises from the use of *Fn* in both broad (*sensu lato*) and strict (*sensu stricto*) senses. *Sensu lato* refers to all historical subspecies of *Fn*, whereas *sensu stricto* denotes the original lineage of *Fnuc* [10]. This confusion underlines the need for a stricter taxonomy.

One further notable example in discussions of *Fusobacterium* taxonomy involves the isolates previously designated as *Fan* C1 [17]. These isolates were linked to *Fwat*, described by Tomida et al. in 2021 [19], which prompted discussion about whether *Fan* C1 and *Fwat* represented the same lineage. Notably, 16S rRNA sequences from *Fan* C1 were publicly available prior to *Fwat* and the majority of *Fan* C1 isolates had already been assigned and deposited in the Korean Collection for Oral Microbiology (KCOM) as *Fan* or *Fn* subsp. *animalis* before the first public release of *Fwat* [20]. Following this, the original authors of both the *Fan* C1 isolates and *Fwat* collaborated to sequence and release related genomes, facilitating an update to the nomenclature [21]. Their results indicate that *Fwat* isolates belong to the “*Fusobacterium nucleatum_J*” group, which also includes strain 13–08-02, FNU (“*F. nucleatum* subsp. *unique*”) [22], and members of the group recently described as *Fan* C1 [10,17,21,23]. Therefore, the name *Fwat*, an already recognized species name under the List of Prokaryotic names with Standing in Nomenclature [24], can now be applied to the group. Currently, *Fwat* seems to be the closest phylogenetic clade to colorectal cancer-associated *Fan* C2. Similarly, *Fusobacterium* strain Vestland19, isolated from a blood culture and recently described by Bivand et al. [25], represents a novel species more closely related to *Fan* than any other known species. Therefore, the authors suggested the name *Fusobacterium paranimalis* sp. nov [11].

Within *Fusobacterium* lineages, ANI values often straddle the 95–96% species demarcation threshold, complicating accurate species assignments [10]. Therefore, according to Goris et al., a 94.5% ANI threshold appears most discerning for *Fusobacterium* species assignments [10,26].

Other species such as *Fusobacterium canifelinum*, *Fusobacterium hwasookii*, and *Fusobacterium simiae*, initially proposed as distinct based on DNA – DNA hybridization and biochemical similarity, are phylogenetically supported as part of *Fn* [10]. For example, *F. simiae* strain W1481 was originally published as a potential novel *Fn* subspecies [27,28]. Accordingly, Zepeda-Rivera et al. suggest, that *Fnuc* should be consistently termed *F. nucleatum sensu stricto*, while lineages within *Fn sensu lato* with unassigned species names (e.g. *F. nucleatum_D*) should be tentatively labeled as *F. unassigned_D*. To avoid ambiguity, they recommend three- or four-letter species abbreviations over two-letter codes [10].

Interestingly, Connolly et al. identified six distinct populations within 133 *Fn* genomes through HGT networks, supporting the described species *Fnuc, Fan, Fpo, Fvn* and *Fwat*. They also identified a previously unreported population, designated *species novel* (*Fusobacterium sp. nov*.), most closely related to *Fnuc* [29].

Overall, these findings reveal substantial diversity within *Fn*, highlighting the need for precise taxonomic frameworks.

## Niche specificity of F. nucleatum populations

Recent work has demonstrated that distinct *Fn* populations occupy specific ecological niches and disease-associated environments [29]. Most populations, except *F. sp. nov*., are detected in both the oral cavity and the gut, and gut-associated *Fn* overall increases with age and disease. *Fvn* is found predominantly in the mouth, whereas *Fan* represents the most prevalent gut population and *Fpo* occurs in both habitats [29]. Although *Fn* rarely colonizes the healthy gut, *Fan* is markedly enriched in CRC [17], type 2 diabetes, and Crohn’s disease, suggesting shared ecological pressures across these conditions. Connolly et al. show that across CRC stool samples, *Fan* and *Fpo* are the two most prevalent and increased populations, with *Fan* found even more frequently in CRC stool than in gingiva. Moreover, not only *Fan*, but also *Fpo*, *Fvn*, and *F. sp. nov*.—are elevated in Crohn’s disease. In ulcerative colitis, only *Fvn* is significantly increased [29]. Simulated metagenomic analyses and an *Fn* HGT network demonstrated that specific environmental contexts harbor distinct, ecologically coherent *Fn* populations [29]. Distinct gene profiles exist between populations: *Fan* carries many clusters absent in *Fwat*, while Fpo differs little from *Fan* despite phylogenetic distance [29]. Niche-specific ecological preferences have also been observed within the oral cavity. In the oral cavity sub- and supragingival plaque commonly contained *Fpo* and *Fvn*, found in more than 50% and 30% of samples, respectively [29,30]. Meanwhile, odontogenic abscesses are strongly enriched for *Fan* [30]. *F*. s*p. nov*. is approximately as common as *Fan* in gingiva. *Fnuc* and *F*wat are less frequent, and no *Fn* population is typically detected in saliva, supporting its role as a plaque-associated organism [29,31]. Of course, *Fn* species have also been associated with other body sites and diseases, but here we focused on the areas most relevant in current research for a concise overview.

## Virulence factors and species specificity in the *F. nucleatum* complex

*Fn* pathogenicity is mediated by several VFs, with recent studies indicating (sub-)species-specific variation that challenges the notion of uniform pathogenesis across all strains [15,16]. Recently, substantial effort has been invested in conducting comprehensive pangenomic analyses of multiple *Fusobacterium* strains to map the distribution of virulence determinants [15,30]. Significant variation in their distribution was observed, with adhesion-related VFs being prevalent across strains, highlighting their importance in *Fn* pathogenicity. In line with this, it was reported that *Fusobacterium* species can be categorized into distinct evolutionary lineages with differing sets of virulence-associated genetic determinants [32].

## Secretion systems of *F. nucleatum* group

**Figure 1.**
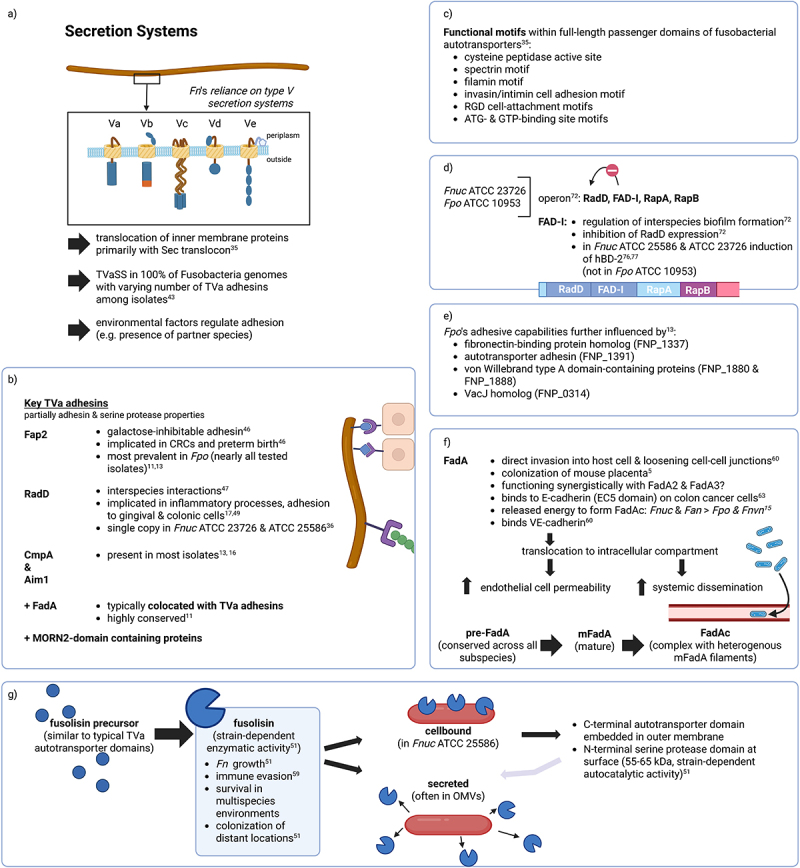
Secretion systems focusing on type Va autotransporters (TVaSS): the figure illustrates the diverse architecture and functional roles of TVSS subtypes (TVa–ve) in Gram-negative bacteria. Section a) provides an overview of TVSSs, b) shows key TVa adhesins, c) functional domains of autotransporters, d) a four-gene operon in *Fnuc* and *Fpo*, e) additional factors influencing adhesion in *Fpo*, f) details about FadA, and g) fusolisin.

Bacterial secretion systems play a crucial role in various cellular functions, including communication, virulence, adhesion, nutrient acquisition, and competition with other microorganisms. In Gram-negative bacteria, these secretion systems have been classified into nine different types (type I – IX) [33]. Some of these systems involve intricate multiprotein complexes that span the bacterial cell envelope, while others, such as the type V secretion system (TVSS), often referred to as autotransporters, adopt a more minimalist architecture. Unlike many bacteria that employ large multiprotein secretion complexes [34, *]Fn* predominantly uses the TVSS [35]. In its simplest form, it typically consists of a single polypeptide chain containing a β-barrel translocator domain embedded in the outer membrane and an extracellular effector or passenger region. Based on the domain organization and functional properties of these proteins, TVSS have been further divided into five subtypes: monomeric autotransporters (TVa and TVd), two-partner secretion systems (TVb), homotrimeric autotransporters (TVc), intimins (TVe) [36], and a recently proposed potential Vf subtype (Figure 1) [33]. Many of the characterized autotransporters function as adhesins or proteases, especially those belonging to the TVa and TVc subtypes [37], facilitating adhesion to extracellular matrix components and other cells, a crucial step in host colonization and biofilm formation [38]. Genomic analyses of *Fnuc* ATCC 25,586 and *Fvn* ATCC 49,256 have revealed a unique reliance on the TVSS, while lacking a type III secretion system, a type IV secretion system, chaperone/usher pathways, and the twin-arginine translocation system [12,35], with the translocation of inner membrane proteins relying primarily on the Sec translocon [12]. Also, the type I secretion system was suggested to be absent contradictory to earlier findings [35]. According to recent findings, genes associated with secretion systems exhibited no clear strain- or (sub-)species-specific patterns, e.g. two genes, *ffh* (potentially involved in protein translocation) and *yadA*, showed identical distributions across strains. Interestingly, *secA* gene, a key ATPase motor protein in the Sec system responsible for preprotein translocation across the cytoplasmic membrane, was predominantly found in *Fpo* [15]. Generally, the translocation of autotransporter proteins through the inner membrane occurs via the Sec apparatus, a protein translocation machinery transporting proteins across the cytoplasmic membrane [35,39,40]. Genomic studies of *Fpo* have further identified a broad range of TVSS (TVa to TVe) next to multiple copies of genes such as *groEL, plr/gapA, tufA*, and *htpB* [13,16]. Meanwhile, in *Fnuc* ATCC 25,586 and *Fvn* ATCC 49,256 only the presence of type Va, Vb and Vc systems was confirmed [35]. Desvaux et al. identified 13 putative autotransporters in *Fnuc* and 11 in *Fvn* [35]. Except for four putative autotransporters originally annotated as serine proteases, all other autotransporters were classified as outer membrane proteins with no additional functional annotations. In line with these observations, Sivertsen et al. highlighted considerable variability in TVSS abundance across the *Fn* group. Their analysis points to extensive diversification within autotransporter gene families annotated simply as “autotransporter domain-containing protein” with numerous loci. This complexity makes orthology and paralogy assignments challenging [11]. Desvaux et al. were also able to identify significant functional motifs within the full-length passenger domains with FN1449 as signature for a cysteine peptidase active site, spectrin repeats, and filamin repeat motifs and FN1893 for invasin/intimin cell adhesion motifs. Moreover, RGD cell-attachment motifs (PS00016), a short amino acid sequence composed of arginine (R), glycine (G), and aspartic acid (D), were found in several passenger domains, either in association with invasin/intimin cell adhesion motifs or alone. This is reminiscent of mechanisms observed in *Escherichia coli* pathotypes (e.g. EPEC and EHEC), whereby bacterial attachment to enterocytes mediated by intimin leads to intestinal epithelial disruption. RGD motifs mediate adhesion to mammalian cells via interactions with β1 integrins [35,41]. Although RGD motifs are often occurring without evidence of functionality, they are frequently associated with cell adhesion functions in autotransporters [42]. Moreover, several ATP- and GTP-binding site motifs were detected in the passenger domains of fusobacterial autotransporters, though no definitive function was attributed.

TVSS proteins usually comprise a β-barrel translocator domain located in the outer membrane and an extracellular passenger domain that is responsible for adhesion or proteolytic activity (a). Key *Fn* adhesins, including Fap2, Aim1, RadD, and CmpA, are shown to mediate cell-to-cell interactions, biofilm formation and host colonization. Additionally, MORN2 domain-containing proteins are depicted, which may contribute to interspecies competition and pathogenicity (b). In the top right-hand corner, functional motifs found in the full-length passenger domain of fusobacterial autotransporters are summarized (c). The four-gene operon that encodes RadD, FAD-I, RapA, and RapB is also depicted (d). This operon was identified in *Fnuc* and *Fpo* (ATCC 23,726 and ATCC 10,953). Further adhesins are described below (e, f). The figure also illustrates the multifunctional serine protease fusolisin, which plays a role in immune evasion and nutrient acquisition (g). The amino acid sequences of the putative serine protease can either remain intact and cell-bound, or the catalytic domain may self-cleave at the peptide bridge connecting both domains, releasing the 55–62 kDa protease. This is secreted and can often be found in outer membrane vesicles.

This figure was created in BioRender.

Interestingly, many autotransporters lack conserved autochaperone domains, indicating variability in their folding mechanisms [35]. Genetic diversity is influenced by recombination and HGT events [16]. While analysis of housekeeping genes (*gyrB, recA, ruvB, ftsZ*, and *rsml*) revealed that genes from *Fnuc, Fan*, and *Fvn* clustered separately from those in *Fpo*, suggesting that recombination events were rare in these housekeeping genes, there is strong evidence for HGT in adhesin-encoding genes between different *Fn* species acting as both donors and recipients, impacting the evolution and adaptation of these adhesins [16].

### Type Va secretion systems

TVaSS are highly present in *Fusobacterium* genomes as shown by early bioinformatic analyses [43]. When analyzing TVa autotransporter homology groups, with representative TVa proteins from *Fnuc* ATCC 23,726 serving as references, 15 distinct TVa protein groups were identified, forming networks with known adhesins [16]. Also, Sivertsen et al. reported, that TVaSS in *Fusobacterium* spp. are not easily classified into discrete groups [11,36,44,45]. The number of TVa adhesins can vary significantly both among and within species [11,16]. It was observed that TVaSS proteins seem enriched in *Fan, Fvn, F. hwasooki*, and in *Fnuc* compared to other species [11].

Key TVaSS adhesins in *Fn* include fusolisin, a serine protease that damages host tissue and inactivates immune effectors, Fap2, Aim1, RadD, and CmpA, each contributing to different aspects of coaggregation, cell-cell interactions, and biofilm formation in both healthy and pathogenic states [16]. Their exact role in carcinogenesis remains unclear. Recent findings indicate that none of these canonical VFs are significantly associated with *Fan* compared to other *Fn* species, suggesting that additional, yet unidentified genetic factors may drive *Fan* enrichment in CRC [17]. The newly recognized *Fwat* lacked *fap2*, *cmpA,* and *fusolisin* and, by *rpoB* gene analysis, these *Fan* strains seemed to form a distinct clade [17]. Fap2, the most prevalent TVa adhesin in *Fn* with high conservation across *Fn* species, is a large galactose-inhibitable adhesin implicated in colorectal cancer (CRC) carcinogenesis and preterm birth as shown in a mouse model [46]. Analysis by Sivertsen et al. of oral and CRC strains from *Fpo, Fan, Fvn, Fnuc, F. hwasooki, F*. sp. W1481, *Fusobacterium canifelinum, Fusobacterium* sp. oral taxon 370, *Fusobacterium pseudoperiodonticum*, and *Fusobacterium periodonticum* revealed that *fap2* was present in 17 of 24 *Fan* strains, 7 of 13 *Fvn* strains, all but one *Fnuc* strains, nearly all *Fpo* strains, and in all other examined strains, highlighting its widespread but variable distribution across *Fusobacterium* species [11]. Similarly, among TVa proteins in *Fpo* strains, Fap2 was shown to be the most prevalent in the *Fpo* genome present in nearly all investigated, though varying in size [13,16]. RadD and CmpA, while smaller, are critical for interspecies adherence and multispecies biofilm architecture [47]. RadD mediates interactions with a diverse set of bacteria and clusters more closely with adhesins from *F. varium* and *F. ulcerans* rather than Fap2 [36,48]. Recently, it was found that RadD is also involved in the cellular attachment with colonic cells [49] and is probably also involved in adhesion to oral gingival keratinocytes [17]. Interestingly, Sivertsen et al. also showed that *radD* was found in 13 of 24 *Fan* strains, all but one *Fvn* strain, 5 of 7 *Fnuc* strains, and in the single investigated *F*. sp. oral taxon 370, but was missing in most *Fpo* strains as well as in the examined *F. pseudoperiodonticum, F. periodonticum, F. hwasooki, F*. sp. W1481, and *F. canifelinum* strains. *Aim1* was detected in 7 of 24 *Fan* strains, 4 of 13 *Fvn* strains, and all but one *Fnuc* strain, while being absent from most *Fpo* strains. Moreover, it was present in the single investigated *F*. sp. W1481 and *F. canifelinum* strains and partially present in *F. hwasooki, F. pseudoperiodonticum*, and *F. periodonticum*, but absent from the *F*. sp. Oral taxon 370 isolate. *CmpA* showed broader prevalence, being present in most *Fan* strains, all but one *Fvn* and *Fnuc* strains, 10 of 26 *Fpo* strains, nearly all *F. pseudoperiodonticum*, 4 of 6 *F. hwasooki*, and in the single examined strains of *F*. sp. W1481, *F. periodonticum*, and *F. canifelinum* [11]. Similarly, in other studies CmpA and Aim1-like adhesins also appeared in most *Fpo* isolates, wheres RadD was the least common [13,16]. These results suggest a high conservation of *radD* and *aim1* across all *Fn* species besides *Fpo* and a generally high conservation of *cmpA* in all *Fn* species. In *Fnuc* ATCC 23,726 and type strain ATCC 25,586 the RadD-gene was present in only a single copy [36]. In *Fpo* isolates lacking *radD*, the entire operon for *radD* was missing while synteny of the surrounding genomic region remained conserved. Wu et al. reported that canonical virulence genes including *fadA3, fap2, fplA, cmpA*, and *fusolisin* are widely distributed across lineage 1 *Fusobacterium* species (especially being associated with CRC), whereas *fadA2, radD*, and *aim1* were rather present in certain non-nucleatum lineage 1 strains. These findings suggest a niche-specific role for these genes, with *fap2, cmpA*, and *fusolisin* contributing primarily to adaptation in the gastrointestinal tract, while *fadA2, radD*, and *aim1* appear more relevant for colonization and survival within the oral environment [11,32,50].

Further studies revealed that *Fnuc* ATCC 23,726 encodes for 25 autotransporters classified as adhesins, serine proteases, or proteins of unknown function. A newly identified TVaSS autotransporter based on the *Fnuc* ATCC 25,586 genome was also reported [36,51]. Notably, RadD in *Fnuc* ATCC 23,726 clusters with serine proteases rather than Fap2 or Aim1. In addition to their adhesive properties, some TVa proteins exhibit serine protease activity, resembling fusolisin, a known virulence-associated protease critical for tissue damage and immune evasion [16,51]. It was shown that *Fnuc* and *Fpo* can secrete the serine protease capable of degrading extracellular matrix proteins, leading to the destruction of periodontal connective tissues and the production of immunoglobulins and complement from the host immune system [18,51,52].

Fusolisin is derived from a precursor protein with domains typical of type Va autotransporters [51,53–55] and can either remain cell-bound or be secreted, depending on the strain with varying enzymatic activity between strains [51]. Membrane-bound, the C-terminal autotransporter domain is embedded into the outer membrane and the N-terminal serine protease domain is transferred to the surface. In most *Fn* strains, the 55–65 kDa catalytic domain cleaves itself after crossing the membrane. However, in strain ATCC 25,586, this autocatalytic activity is less efficient, leading to a full-length, membrane-anchored protease. Interestingly, the presence of serine protease inhibitors can inhibit *Fn* growth, supporting fusolisin’s nutritional role [51,56–58]. At least in strains ATCC 10,953 (isolated from human cervico-facial lesion), and ATCC 23,726, amino acid sequences of the putative serine protease can either remain intact and cell-bound, or the catalytic domain may self-cleave at the peptide bridge connecting both domains, releasing the 55–62 kDa protease. This is secreted and can often be found in outer membrane vesicles, suggesting that the autotransporter domain is not essential for adherence of the catalytic domain to the bacteria’s outer membrane surface. Interestingly, in strains ATCC 23,726, ATCC 10,953, and FDC 364, the full-length (~96 kDa) protease was primarily detected in outer membrane vesicle preparations and being predominantly membrane-bound, while the 56–62 kDa protease was found in the growth media [39]. Notably, fusolisin is not only involved in immune evasion [59] but also likely plays a role in *Fn’s* survival in multispecies environments like the oral cavity and potentially in the placenta where it is plausible that fusobacterial proliferation is fusolisin dependent. Thus, inhibition of fusolisin could impair the bacterium’s ability to colonize distant locations, such as the placenta, underlining its significance in pathogenicity [51]. Sivertsen et al. could find at least one copy of the *fusolisin* gene in nearly all investigated *Fusobacterium* species, indicating a very high conservation [11].

### Type Va scretion system–related virulence factors

FadA, a well-characterized virulence factor, frequently co-locates with TVa adhesin genes. Functionally, FadA mediates attachment to and invasion of host epithelial cells, and its ability to disrupt the gingival epithelial barrier may provide a route for oral strains to disseminate to extraoral sites [36,60–63]. FadA is unique to *Fn* and highly conserved among its strains but absent in non-oral fusobacteria [11,64]. While mutations did not alter its monomeric or dimeric 3D structure, they likely affect binding surface stability and interactions with host receptors [15,61,62,65,66]. These variations may influence the mode of FadA’s combination with host receptors, ultimately impacting *Fn´*s ability to adhere to and invade host cells. It was shown that FadA binds to the EC5 domain of E-cadherin on colon cancer cells [45], with (sub-)species-specific mutations influencing binding efficiency. Notably, the main structure of the precursor FadA (pre-FadA) was conserved across all *Fn* species, despite variations in amino acid sequences at multiple sites. FadA operates through the combination of pre-FadA and mature FadA (mFadA) to form a pre-FadA-mFadA complex (FadAc), which subsequently binds to the host receptor E-cadherin [45,67,68]. While long and thin mFadA filaments alone lack cell-binding activity, heterogeneous filaments formed by the FadAc enable host cell attachment [69]. Notably, even with multiple mutation sites in different *Fn* species, the binding mode of pre-FadA and mFadA remained consistent. Among *Fn* subspecies, *Fan* and *Fnuc* released more binding free energy while forming the FadAc complex than *Fpo* and *Fvn*, whereas *Fvn* exhibited the largest interface area and lowest binding free energy, suggesting unique interactions [15]. Binding free energy describes the strength of the interaction between, e.g., adhesine and receptor. It represents the free energy difference between the bound and completely unbound states. FadA allows both direct invasion into host cells and pericellular invasion by loosening cell-cell junctions. It also binds vascular endothelial cadherin (VE-cadherin), translocating from the cell-cell junctions to intracellular compartments, thereby increasing endothelial cell permeability and promoting systemic dissemination [60].

Earlier analyses of *fadA* distribution across a limited set of *Fusobacterium* genomes suggested that *fadA* is absent in passively invading species and more common in highly invasive ones [45]. However, this pattern does not consistently align with observations from in vivo invasion assays [36,45,61,70]. Genes encoding secreted FadA are broadly conserved across strains [71]. Researchers identified two homologous genes, *fadA2* and *fadA3*, with FadA3 being specifically implicated in host cell binding and invasion in *Fpo* ATCC 12,230 [36]. In other strains it still has to be fully characterized. FadA2, also called RapA, is very similar to FadA, thus it could be a second form of FadA, though functional characterization of RapA remains lacking [36]. However, their absence did not have a consistent significant impact on *Fn* behavior in either species tested [72]. It remains unclear whether FadA2 and FadA3 function as adhesins in a manner similar to FadA but with distinct, unidentified host receptor molecules. The secretion of FadA is at least partly mediated by the autotransporter Fap2, though since Fap2 is only present in some strains, other TVaSS may also facilitate its transport across the cell wall [11]. Broad genomic surveys confirm that *fadA* is present in all investigated strains across multiple *Fusobacterium* species, while *fadA2* showed a heterogeneous distribution. Thus, it was present in approximately 50% of investigated *Fan* strains as well as in all *Fvn* and *Fnuc* strains, largely absent from *Fpo* except for two strains carrying four copies of the gene, detected in a single examined *F*. sp. oral taxon 370 isolate, and absent from investigated *F. pseudoperiodonticum, F. periodonticum, F. hwasooki, F*. sp. W1481, and *F. canifelinum* strains. In contrast, fadA3 was highly prevalent across nearly all strains surveyed and typically present in multiple copies, indicating its broad conservation and potential functional importance [11]. Another study showed that gene triplication of *fadA3* was observed in *Fnuc* ATCC 23,726 and *Fnuc* ATCC 25,586, but not in *Fpo* ATCC 12,230. Similarly, *Fnuc* ATCC 23,726 contained four *fadA* orthologs, while *Fpo* ATCC 12,230 encoded only one, alongside at least one additional *fadA* paralog, indicating that different (sub-)species likely employ distinct adhesins for host interactions [18,61,73]. Although highly conserved, the nucleotide and amino acid sequences of *fadA* and also *fplA* segregate by *Fn* species, potentially reflecting differential interactions with host ligands. According to Zepeda-Rivera et al., *Fwat* showed enrichment of *fadA2* together with other adhesins such as *radD* and *aim1*, which may be particularly important in the oral niche, and lacked *fap2, cmpA*, and *fusolisin*. In contrast, these three factors were significantly associated with *Fan*, which exhibited increased adherence and invasion in CRC models [17]. Interestingly, the region between *fadA* and *fap2* displays considerable variation, particularly in the repertoire and copy number of TVa adhesins. Phenotypic analyses of *Fpo* isolates revealed significant variation in adhesion to oral keratinocytes, ranging from 5.5% to 51% [16]. Strains possessing multiple TVa adhesin genes generally exhibited higher adhesion rates, although a direct correlation between copy number and adhesion is not always observed. Notably, *fadA* gene copy number does not appear to significantly impact adhesion to this specific cell line. Additionally, FadA plays a crucial role in the colonization of *Fpo* ATCC 12,230 in the mouse placenta [5] and was also shown to be a key driver of host cell signaling in this strain. Interestingly, *Fnuc* ATCC 23,726 possesses five FadA family proteins, raising questions about whether they function synergistically during infection or if FadA, FadA2, and FadA3 have distinct roles.

Beyond FadA and TVa adhesins, other surface proteins contributing to *Fpo‘*s adhesive capabilities were identified. These include a fibronectin-binding protein homolog (FNP_1337), an autotransporter adhesin (FNP_1391), and two von Willebrand type A domain-containing proteins (FNP_1880 and FNP_1888). Additionally, a VacJ homolog (FNP_0314), known for its role in the intracellular spread of *Shigella flexneri*, may play a role in epithelial invasion and systemic dissemination of *Fpo* [13].

Using the type strains *Fnuc* ATCC 23,726 and *Fpo* ATCC 10,953, a four-gene operon encoding RadD, FAD-I, RapA, and RapB was identified, probably also playing a crucial role in adhesion and biofilm formation [72]. Mutants lacking FAD-I, RapA, or RapB exhibit increased binding to *Streptococcus gordonii*, with the Δ*fad-I* mutant displaying the most pronounced effect. FAD-I regulates *radD* expression, thus the increased binding was primarily triggered by FAD-I-mediated overexpression of *radD*, leading to a denser and thicker biofilm [72]. This suggests that FAD-I plays an essential regulatory role in the formation of interspecies biofilms. These findings also reinforce the hypothesis that adhesin regulation is tightly controlled in *Fn*, and it is likely influenced by environmental factors, such as the presence of partner species. This regulation is essential for bacterial survival in the fluctuating environments of the oral cavity, where the ability to form stable biofilms and interact with other species plays a key role in pathogenicity. Moreover, RadD also contributes to lymphocyte apoptosis and strain-specific binding to *Porphyromonas gingivalis* [74,75]. Additionally, FAD-I has been implicated in the induction of human β-defensin 2 (hBD-2) upon contact with oral epithelial cells in a species-dependent manner [76,77]. While FAD-I from *Fnuc*, type strains ATCC 25,586 and ATCC 23,726, induced expression of hBD-2, FAD-I from *Fpo*, type strain ATCC 10,953, did not demonstrate this ability. Small sequence variations in key proteins could explain these differences in immune modulation induced by FAD-I.

Researchers could further find a frequent and extensive enrichment of MORN2 (membrane ontology and recognition nexus type 2) domain-containing proteins consisting of 1 to 27 repeats of a ~ 20-amino-acid domain. This expansion is highly specific to *Fusobacterium* [45]. The exact function of MORN2 domain-containing proteins remains unknown, but most possess signal sequences indicating potential export to the periplasmic space, the outer membrane, or even secretion into the extracellular environment. MORN2 domain proteins are enriched in invasive *Fn* species and are frequently found in genomic regions containing TVa autotransporter genes. Some have been identified as part of the “FusoSecretome” [36,78]. MORN2 domain-containing proteins are secreted into *Fn* outer membrane vesicles [79] and possess YwqK domains, which may function as toxin-antitoxin systems for interspecies competition or as bacterial abortive infection systems that limit viral replication, potentially being activated by phage infection [80]. Recent findings further suggest that alternative adhesins and invasion mechanisms like MORN2 May compensate for the absence of FadA family proteins in certain *Fusobacterium* strains [36]. Collectively, autotransporters, FadA, and MORN2 proteins likely form a docking and invasion network critical to host interactions and pathogenicity [36].

### Type Vb secretion systems

**Figure 2.**
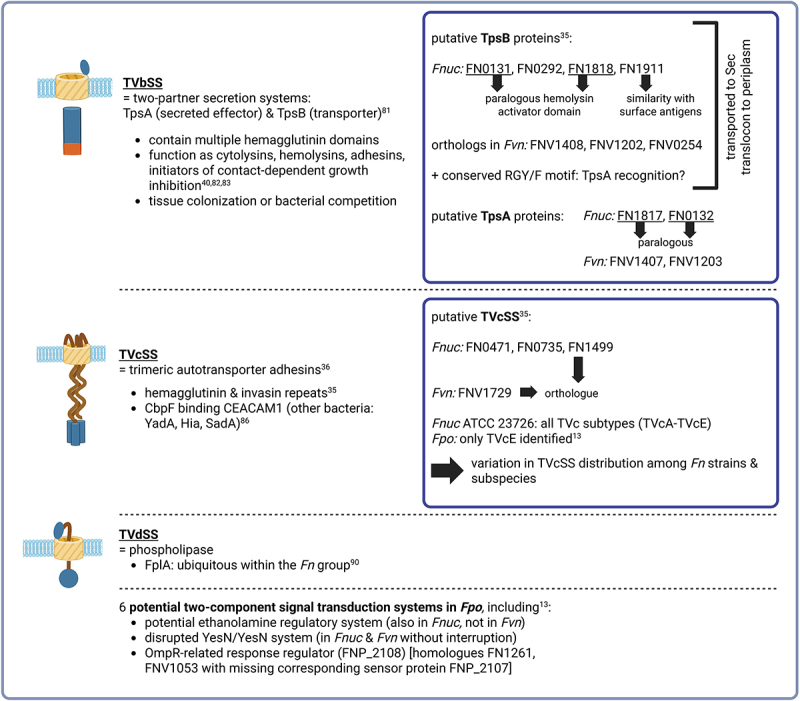
Further type V secretion systems.Type Vb secretion systems (TVbSS, two-partner secretion): TVbSS consist of two proteins: TpsA, the secreted effector, and TpsB, the dedicated transporter. These proteins function in adhesion, cytolysis, hemolysis and contact-dependent growth inhibition. Four putative TpsB proteins (FN0131, FN0292, FN1818 and FN1911) were identified in *Fnuc*, with FN0131 and FN1818 containing hemolysin activator domains and FN1911 showing similarity to surface antigens. The conserved RGY/F motif likely mediates TpsA recognition. Genes encoding TpsA and TpsB are usually found together in operons. These proteins may contribute to tissue colonization, bacterial competition and immune modulation, including the induction of apoptosis in immune cells.

Type Vb Secretion Systems (TVbSS), also known as two-partner secretion systems (Figure 2), typically involve two proteins: TpsA (secreted effector) and TpsB (dedicated transporter) [81]. This separation allows for greater flexibility in the fate of the secreted protein, with some remaining attached to the outer membrane while others are fully released into the extracellular environment. TVbSS proteins contain multiple hemagglutinin domains and function as cytolysins, hemolysins, adhesins, and proteins that initiate contact-dependent growth inhibition to compete with neighboring bacteria [35,36,40,82,83]. In *Fn* TVbSS displayed less variation, with most isolates carrying between one and three copies in *Fpo* [13]. In *Fnuc*, four putative TpsB proteins have been identified: FN0131, FN0292, FN1818, and FN1911. FN0131 and FN1818 are paralogous (arisen through gene duplication within *Fnuc*), while *Fvn* contains their orthologues FNV1408, FNV1202, and FNV0254 (arisen through speciation of *Fvn*). These TpsB proteins are transported to the periplasm via the Sec translocon. Full-length *Fnuc* TpsB proteins revealed the presence of a hemolysin activator domain within FN0131 and FN1818, further supporting a role for these proteins in the type Vb secretion pathway, while FN1911 shares similarity with surface antigens [35]. Moreover, a conserved RGY/F motif, possibly involved in TpsA recognition, was consistently present in these proteins. Notably, no covalent link exists between TpsA and TpsB, suggesting that a specific recognition event must occur at the periplasmic side of the outer membrane prior to translocation of TpsA to the bacterial cell surface [84]. TpsA proteins bear a conserved and highly distinctive N-proximal domain called the TPS domain, essential for secretion and hypothesized to interact specifically with TpsB to initiate translocation. FN1817 and FN0132, both paralogous TpsA proteins, were identified in *Fnuc* with orthologues FNV1407 and FNV1203 in *Fvn*. In *Fn*, most TpsA proteins are annotated as hemolysins, containing hemagglutinin repeats functioning as hemagglutination activity domains. Genes encoding TpsA and TpsB are generally organized consecutively in an operon [35]. TVbSS in *Fusobacterium* could be involved in either tissue colonization or bacterial competition. Among the strains studied to date, *Fnuc* ATCC 23,726 encodes the largest number of functional genes associated with this system [36]. Unlike many pathogenic bacteria, *Fn* does not produce active secreted toxins. However, its proteins may function as uncharacterized effectors, influencing host immune responses. For instance, *Fn* has been shown to induce apoptosis in peripheral blood mononuclear cells, hinting at potential immune-modulatory roles for its secreted proteins [48,85].

### Type Vc secretion systems

Type Vc Secretion Systems (TVcSS), also known as trimeric autotransporter adhesins (Figure 2), form long fibrous structures extending up to over 100 nm from the bacterial surface, enabling adhesive interactions with host cells [36]. The TVcSS is a subfamily of surface-attached oligomeric autotransporters, also referred to as AT-2 proteins. Like classical autotransporters, they are translocated across the inner membrane via the Sec apparatus and subsequently anchored in the outer membrane by their C-terminal regions. Fusobacterial AT-2 proteins often possess hemagglutinin and invasin repeats [35]. In *Fn*, the most notable TVcSS protein is CbpF, which has demonstrated the ability to bind to CEACAM1 on human cells [86]. CbpF is part of a larger family of TVcSS that includes VFs from other pathogens, such as YadA from *Yersinia* spp., Hia from *Haemophilus influenzae*, and SadA from *Salmonella enterica* [37,87–89]. Using the C-terminal region of YadA as a reference, three putative AT-2 proteins (FN0471, FN0735, FN1499) were identified in *Fnuc*. FN1499 has an orthologue (FNV1729) in *Fvn*, while FN0471 and FN0735 lack orthologues in *Fvn*. These proteins contain signal peptides, translocation units, and passenger domains. Desvaux et al. reported that several hundred homologues related to invasins, immunoglobulin-binding proteins, serum resistance proteins, or haemagglutinins have been identified [35]. Interestingly, while *Fnuc* ATCC 23,726 possesses all subtypes of TVc autotransporters (TVcA – TVcE), only TVcE was identified in *Fpo*, typically in a single copy [13]. Interestingly, *Fwat* is enriched in T5cSS relative to other species, another feature distinguishing it from *Fan* [11]. This variation in TVcSS distribution among different *Fn* species highlights the potential for diverse adhesion and virulence capabilities within *Fn*. Although TVcSS proteins are crucial for bacterial adhesion, their role in cellular invasion remains unclear and requires further investigation [36].

### Type Vd secretion systems

While a striking overall variability in both the abundance and distribution of TVSS types was observed, it was reported that only one TVdSS component – FplA [90]—appeared to be present and showed only little variation across strains. FplA is a membrane-anchored phospholipase A1 enzyme composed of a patatin domain (phospholipase activity), a POTRA domain (protein – protein interactions), a C-terminal β-barrel (outer membrane insertion), and a unique N-terminal extension that likely enhances catalytic efficiency by supporting proper folding and active site positioning. FplA binds with high affinity to host phosphoinositide signaling lipids, suggesting a role in establishing an intracellular niche [90].

Generally, all TVSS play a crucial role in *Fn* pathogenesis, contributing to adhesion, immune modulation, and tissue invasion. An overview is given in Figures 1 and 2. Notably, several type V secreted adhesins have also been linked to IL-8 induction, a key factor in periodontal disease progression [35,91]. Moreover, *Fnuc* harbors a cysteine protease autotransporter, the first of its kind reported, with structural features suggesting interactions with cytoskeletal proteins. Given the role of cysteine proteases in *P. gingivalis*—where they degrade fibrin, increase vascular permeability, and suppress immune responses [92,93]—*Fn* may employ a similar mechanism to evade host defenses and contribute to periodontitis progression [35]. Additionally, *Fn* also contains serine protease autotransporters of the subtilase family, which likely play a role in TpsA maturation. One of these proteases degrades extracellular matrix components, potentially facilitating tissue invasion [59], while several autotransporters have also been implicated in degrading antibacterial serum components, reinforcing their role in immune modulation. Furthermore, the phase variation observed in TpsA and TpsB gene expression suggests an adaptive mechanism that allows *Fn* to regulate VF production in response to environmental cues, a strategy frequently associated with immune evasion [35,94].

Type Vc secretion systems (TVcSS, trimeric autotransporter adhesins): Three putative TVcSS were identified in *Fnuc*: FN0471, FN0735, and FN1499. FN1499 (CbpF) binds to human CEACAM1.

Type Vd secretion systems: These are present as single-copy phospholipase autotransporters that are conserved across *Fpo* isolates.

Furthermore, *Fpo* harbors several two-component signal transduction systems, which are summarized in the lower part of the figure.

This figure was created in BioRender.

Research further revealed six potential two-component signal transduction systems in *Fpo*, three of which stand out due to their unique genetic organization. These include a potential ethanolamine regulatory system also prevalent in *Fnuc* but not *Fvn*, a disrupted YesM/YesN system, which is encoded without interruption by *Fnuc* and *Fvn*, and an OmpR-related response regulator (FNP_2108) with a distinct signaling pathway suggesting functional divergence among *Fn* strains. Notably, FNP_2108 has homologs in previously sequenced *Fn* genomes (FN1261, FNV1053) [13], although the corresponding sensor protein (FNP_2107) activating OmpR is absent in these genomes. Omp85-like proteins, at least encoded by *Fnuc*, are related to the TpsB family, though their precise role in outer membrane protein assembly remains debated [35,95–98]. Overall, *Fn* employs a diverse arsenal of TVSSs, proteases, and regulatory pathways to establish itself in host tissues, evade immune responses, and contribute to several diseases.

## Type IV conjugal pili of F. nucleatum group

**Figure 3.**
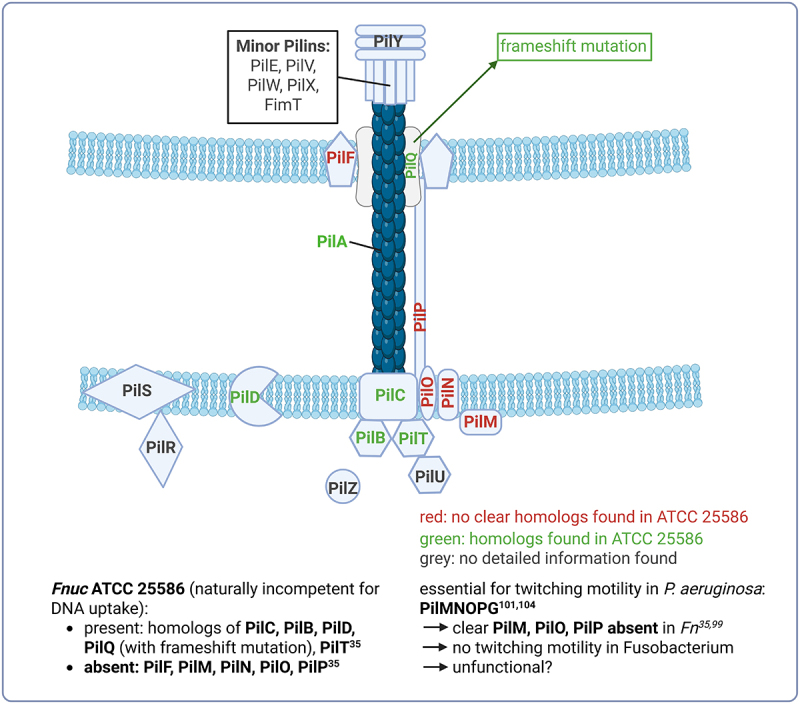
Type IV conjugal pilus (Tfp) system: the figure shows a Tfp system consisting of several Pil proteins. The Tfp machinery facilitates the initial attachment of bacteria through pilus fibers, followed by stable association via surface proteins. In addition to adhesion, tfps can enable twitching motility through pilus extension and retraction. However, *Fn* lacks several essential pilus assembly genes (*pilM, pilO,* and *pilP*), highlighted in red, and exhibits a frameshift mutation in *pilQ* in *Fnuc* ATCC 25,586. This is likely to render its Tfp nonfunctional for twitching motility and natural competence. Nevertheless, homologues of key Tfp components (PilB, PilC, PilD, PilQ and PilT) are present, suggesting the existence of a minimalist or divergent Tfp system. The present homologs are highlighted in green.

The type IV conjugal pili (Tfp, f = four) machinery is a conserved system within the *Fusobacterium* genus (Figure 3) [99]. Bacterial adhesion typically involves an initial attachment via pili, followed by a more intimate association through surface proteins [100]. Tfp, present in organisms such as *Pseudomonas aeruginosa* and *Neisseria gonorrhoeae*, facilitates colonization and biofilm formation through interactions with inert surfaces, bacterial cells, and mammalian cells [101,102]. The attachment of Tfp to inert surfaces occurs via nonspecific adhesion at the pilus tip, whereas binding to eukaryotic cells involves specific interactions with carbohydrate receptors. Beyond adhesion, Tfp mediates twitching motility, a form of surface translocation dependent on alternating extension and retraction of the pilus filament [101]. In Gram-negative bacteria, a pilus composed of pilin proteins is fed through an outer membrane pore formed by PilQ (for pilus deployment), followed by retraction into the periplasm. During this process, DNA is unwound into single-stranded DNA (ssDNA) and delivered to the cytoplasmic membrane channel (ComEC/ComA) and other cytoplasmic proteins that are present in all known competent bacteria [103]. Comprehensive genome sequence analyses of *Fnuc* ATCC 25,586 and *Fvn* ATCC 49,256 have revealed the presence of a Tfp locus [99]. Tfp may contribute to the lectin-like adherence patterns observed in *Fn*. The Tfp locus could also be responsible for the polarly localized pili observed via electron microscopy. *Fn* contains two nucleotide-binding proteins homologous to PilB and PilT. While both Tfp and type II secretion systems require PilB homologs for protein secretion, PilT homologs, which are essential for pilus retraction, are unique to Tfp systems [101]. The operonic structure of genes encoding PilA, PilC, PilN, and PilQ homologs, along with several prepilin-like proteins, suggests that *Fn* may be capable of assembling Tfp. However, the *pilMNOPQ* genes, which are partially absent in *Fnuc* ATCC 23,726 [99], have been shown to be essential for twitching motility and pilus assembly in *P. aeruginosa* [101,104]. This could explain why *Fusobacterium* does not display twitching motility. The absence of clear *pilM, pilO*, and *pilP* counterparts may indicate mutational attrition, potentially rendering Tfp nonfunctional. Interestingly, some bacteria retain functional Tfp systems despite lacking PilP, as observed in *Synechocystis* species. Recent genomic analyses of *Fnuc* ATCC 25,586 found homologs of PilB, PilC, PilD, PilQ, and PilT, all traditionally involved in Tfp formation and function [35]. This raises the question of whether these genes are components of a type IV protein secretion system, a Tfp-facilitated natural competence system, or a type II protein secretion system, warranting further investigation. Despite possessing genes associated with the type IV pilin/fimbriation system, *Fn* appears to have a minimalist system, missing several genes commonly deemed essential for natural competence in other bacteria. Intriguingly, absent are PilF, an outer membrane lipoprotein crucial for the biogenesis and localization of the secretin, as well as PilM, PilN, PilO, and PilP, which together form the inner membrane pilus subcomplex. This subcomplex is essential for aligning the outer membrane secretin with the pilus assembly machinery [104]. Nevertheless, *Fn* does not appear to require these components for exogenous DNA transfer via its Tfp-facilitated natural competence system. Further investigation revealed that the *pilQ* gene in *Fnuc* ATCC 25,586 contains a frameshift mutation that disrupts its integrity, potentially preventing the formation of functional pili required for DNA import. This frameshift renders the strain naturally incompetent for DNA uptake, as experimentally confirmed. Sanders et al. demonstrated that the deletion of *pilQ* and *comEC* (for cytoplasmic DNA import) in *Fnuc* ATCC 23,726 abolished DNA uptake and chromosomal incorporation in this strain. These findings underscore the essential roles of PilQ and ComEC in the competence process, highlighting their necessity for the successful import and chromosomal integration of exogenous DNA [99]. Notably, DNA-uptake efficiency varied between ATCC strains. It remains unclear whether this capacity extends to the other, comparatively understudied *Fusobacterium* species.

## Metabolic features of F. nucleatum group

*Fn* species exhibit marked variations in metabolic features that likely underpin its differing niche adaptations and disease associations. In particular, phospholipid- and carbohydrate-derived nutrient utilization, iron acquisition, and amino acid biosynthesis emerge as key discriminating features [17,29,30,32].

### Ethanolamine and 1,2 propanediol utilization operons

Ethanolamine (EA; *eut* operon) and 1,2propanediol (1,2PD; *pdu* operon) utilization enhances gastrointestinal niche adaptation by expanding metabolic capacity for EA and 1,2PD utilization. ZepedaRivera et al. showed that in *Fan*, exposure to EA or 1,2PD induced both operons transcriptionally and upregulated 13.02% of *Fan*-associated genes, whereas *eut* and *pdu* are genetically absent in *Fwat*. Notably, *radD* and *aim1*, although present in both species, were selectively upregulated in *Fan* in the presence of EA, but not in *Fwat*. Further VFs encoded by *Fan* were also induced: *cmpA, fusolisin* and *fap2* were upregulated in response to EA, and *fap2* was additionally induced by 1,2PD. These data support that presence of EA and 1,2PD in the gastrointestinal tract triggers a virulenceassociated transcriptional programme in *Fan*, promoting adaptation to and persistence within extraoral niches such as CRC tumors [17]. *Eut* and *pdu* operons have been strongly implicated in the CRC affinity of *Fan* [17]. Wu et al. reported that the *eut* and *pdu* operons are broadly distributed within CRC associated lineage 1 (L1) of *Fusobacterium* species consisting of *Fn* species, *F. periodonticum*, *F. pseudoperiodonticum*, *F. hwasookii* and many closely related species [32]. While the *eut* operon is highly conserved across *Fn* species besides *Fwat* [11,17,32*], pdu* operons were only found in *Fan* [17, *]Fpo*, *F. canifelinum, F. hwasookii* and strain KCOM 2305 of *F. pseudoperiodonticum* [32]. Consistent with these findings, comparative genomic analysis indicates that *pdu* is highly conserved in *Fpo* and *F. hwasookii*, present in almost all *Fan* strains but absent from *Fnuc* and *Fvn* [11].

### Acid stress resistance and gastric transit

Successful colonization of distal gastrointestinal sites requires survival of gastric acid. Being conserved across all examined *Fan* genomes but absent from *Fwat*, the glutamate-dependent acid resistance (GDAR) system was suggested to enable the survival of *Fan* during gastric passage and to support growth in the mildly acidic to neutral environments typical of the lower gastrointestinal tract, thereby facilitating direct descent through the gut lumen and subsequent infiltration of CRC [17]. GDAR is among the most potent acid resistance mechanisms described in gut bacteria, and requires only glutamate to function at pH ≤ 3 [17]. It relies on glutamate decarboxylase enzymes (GAD) that convert intracellular glutamate into γaminobutyric acid (GABA), a reaction that consumes one cytoplasmic proton and thereby helps keep the cytosol less acidic [105].

Furthermore, in a study using seven *Fusobacterium* strains, including *Fnuc* ATCC 25,586 and *Fn* strain 612, *Fn* exhibited the highest survival in a stomach-like acidified fluid at pH 1.5 [106]. This acid resistance was linked to high levels of the monounsaturated fatty acid (MUFA) erucic acid (C22:1(n9)) in the cell membrane, regulated by the FnFabM gene, an enoyl-CoA hydratase – related protein. Generally, shifts in membrane fatty acid composition with increased MUFAs and decreased saturated fatty acids contributed to acid tolerance [106]. Further bacterial stress-response systems, including the two-component systems CarRS and ModRS enhancing interspecies interactions and providing defenses against oxidative stress [16,107] and the extracytoplasmic function sigma factor (ECF) sigma E (σ^E^) and its encoding gene *RpoE* [108] have been suggested to support survival under extreme acid stress.

### Modulation of host redox balance and prooncogenic metabolites

Beyond direct virulence factors, *Fan* exerts profound metabolic effects on the intestinal milieu that favor tumor progression. In a mouse model, metabolomic profiling revealed that *Fan*treatment was associated with significant enrichment in glutathione metabolism and γ-glutamyl amino acid pathways compared to *Fwat*-treated mice. Interestingly, levels of components involved in γ-glutamyl-cysteinyl-glycine (GSH) synthesis changed [17]. Because an elevated oxidized-to-reduced glutathione ratio (GSSG/GSH) sensitizes cells to oxidative stress, inflammation, and tumor progression [109], the increased GSSG/GSH ratio observed in *Fan*-treated mice – relative to control and *Fwat* groups – indicates heightened oxidative stress. Consistently, levels of other oxidative stress markers, such as cystine and cysteineglutathione disulfide, were significantly elevated, while concentrations of polyamines with reactive oxygen species (ROS) – scavenging properties (putrescine, spermidine, and spermine) were reduced. As γ-glutamyl transpeptidase – mediated GSH metabolism can generate ROS and promote genomic instability [17,110], these shifts provide a plausible mechanistic link between *Fan* colonization and the severe chromosomal abnormalities associated with *Fusobacterium* in epithelial cells [111].

Inflammation-associated metabolites were likewise altered in *Fan*colonised mice. Anti-inflammatory polyamines were depleted, alongside increased levels of N-monomethylarginine and dimethylarginine, inhibitors of nitric oxide synthesis.

Pro-inflammatory prostaglandins and ceramides, including prostaglandin A2, Npalmitoylsphingosine, and Npalmitoylsphingadienine, were also elevated, as were eicosanoids linked to cancer growth and metastasis [17,112]. Moreover, the detected increased 6-keto-prostaglandin F1α further implicates COX2-dependent arachidonic acid metabolism, a pathway upregulated in *Fn*-associated human CRC [17,113]. Together, these changes show that *Fan*—but not *Fwat*—drives a pro-inflammatory, pro-oncogenic intestinal metabolic state.

### ABC transporters

Additonally, several ATP-binding cassette (ABC) transporters were identified in *Fn*, facilitating the uptake and efflux of various substrates, including carbohydrates, lipids, proteins, peptides, amino acids, ions, and also antibiotics [12,114]. However, ABC transporters alone cannot mediate protein secretion into the extracellular medium without a membrane fusion protein spanning the inner membrane and periplasm, and an outer membrane factor, such as TolC [115,116]. Searches for membrane fusion proteins (FN0516, FN0826, FN1274) and outer membrane factors (FN1273, FN0517, FNV0260, FNV0611) homologues revealed that all were associated with detoxification efflux systems and belonged to the resistance-nodulation-cell division (RND) superfamily in *Fnuc* ATCC 25,586 and *Fvn* ATCC 49,256 [116]. In *Fpo* several potential drug transporters were identified, including MOP/MATE family (multidrug/oligosaccharidyl-lipid/polysaccharide/multi-antimicrobial extrusion protein) efflux pumps (FNP_0174, FNP_0640, FNP_0890, FNP_1162, FNP_1207, FNP_1299, FNP_1596), DMT superfamily (drug/metabolite) transporters (FNP_0388, FNP_0622), and RND family antiporters (FNP_0507, FNP_0508), likely contributing to antibiotic and drug efflux [13,117]. Moreover, four predicted beta-lactamase genes were identified, one of which (FNP_0627) is unique to *Fpo*. A number of 14 outer membrane protein genes were described, with all but one being present in *Fpo*. Notably, the porin FomA (FNP_0972), which was shown to have immune stimulatory properties and plays a role in Toll-like receptor (TLR) 2 signaling [13,118], was detected in *Fpo* but not in *Fvn* [12,117]. Identifying VFs related to effector delivery systems, *clpV* was shown to be present in all strains at a high copy number (seven copies). ClpV is an AAA^+^ (ATPase associated with various cellular activities) protein disassembling the contracted sheath in the type VI secretion system [119]. Other VFs, present in lower copy numbers (one or two copies), were consistently distributed across strains, but their occurrence did not follow a clear (sub-)species-level pattern [15].

### Iron acquisition systems

*Fn* (sub-)species also differ in iron acquisition, with *Fan* specifically enriched for high-affinity ferrous iron transport systems [29]. *Fan* genomes consistently carry gene clusters, including FeoAB system, largely absent in other populations except sporadically in *Fpo* [29,30].

*Fan* retains the core set of iron transport/utilization genes shared with other oral fusobacteria, but the additional high-affinity FeoAB system likely provides a strong growth advantage in iron-restricted, hypoxic or anoxic environments. FeoAB systems, key virulence factors in other pathogens including *Porphyromonas gingivalis*, *Salmonella enterica*, and *Helicobacter pylori* [120–124], may support *Fan* proliferation in necrotic or abscess-like tissues, where reduced ferrous iron is abundant [30,120,125]. FeoA/B has also been proposed as a potential biomarker in oral bacteria for their ability to colonize the gut, given the repeated implication of iron in CRC pathogenesis [120,126].

ABC transporters also play a crucial role in iron acquisiton [12]. Interestingly, in *Fpo* 26 predicted proteins are involved in iron uptake. Notably, proteins *HmuV* (FNP_2267), *HmuU* (FNP_2266), and *HmuT* (FNP_2269) form a heme ABC superfamily transporter. Additionally, two other proteins (FNP_2270 and FNP_1765) are likely TonB-dependent heme receptors. There are also two additional iron ABC transporters (FNP_428-430 and FNP_1451-1454), one cobalamin/iron ABC transporter (FNP_0398–0341), and one Nramp family iron transporter (FNP_1660). *Fpo* also possesses an OfeT family oxidase-dependent iron transporter (FNP_0531), and three hemolysin genes (FNP_0006, FNP_0159, and FNP_0999), with the latter two associated with TPS family two-partner secretion proteins, which is similar to what is seen in *Fvn* but different from *Fnuc* (which has three such pairs) [12,13,117]. Some iron transporters, such as FNP_0339, FNP_0426, FNP_0428, FNP_0531, and FNP_0769, are present in *Fnuc* but missing in *Fvn*, suggesting that *Fvn* might have a reduced iron requirement. Furthermore, a homolog of the ferric uptake regulator, Fur (FNP_2353), is present in *Fpo* but absent in *Fvn* [13,117].

Thus, especially *Fan* and *Fpo*, showing the highest detection in the gut environment [29], exhibit the highest iron acquisiton capacities.

### Amino acid biosynthesis and metabolic independence

While *Fan* is distinguished by iron transport and CRClinked traits, *Fpo* and *F. periodonticum* stand out by their extensive amino acid biosynthetic capacities and broader metabolic independence. Thus, *Fpo* genomes are enriched for biosynthetic gene clusters relative to other *Fn* populations enabling synthesis of amino acids, fatty acids, and nucleic acids, supporting metabolic autonomy and colonization of multiple niches [29,127]. Thus, *Fpo* and *F. periodonticum* encode complete biosynthetic pathways for multiple amino acids, including methionine, lysine, threonine, cysteine, isoleucine, valine and leucine: *ilvC* (Ketol-acid reductoisomerase), *ilvH* (Acetolactate synthase, small subunit), *leuC* (Homoaconitase/3-isopropylmalate dehydratase large subunit), *leuD* (3-isopropylmalate dehydratase small subunit), *thrB* (Homoserine kinase), *cysI* (Sulfite reductase, beta subunit), *thrC* (Threonine synthase), *asd* (Aspartate-semialdehyde dehydrogenase), *metL1* (Aspartate kinase), *metA* (Homoserine O-succinyltransferase), *MET17* (O-acetylhomoserine/O-acetylserine sulfhydrylase, pyridoxal phosphate-dependent) and others not being present in 100% *of Fpo* and *F. periodonticum* [30]. Both organisms additionally encode the ABCtype molybdenum transporter ModF. As molybdenum is a critical cofactor for enzymes for a number of enzymes, but is especially important in pathways involved in various aspects of sulfur metabolism [128], the linkage of *modF* to methionine and cysteine biosynthesis genes suggests tight integration between trace metal acquisition and amino acid production in these species. The combination of expanded biosynthetic repertoires and molybdenum-dependent sulfur metabolism likely underpins the apparent fitness advantage of *F. periodonticum* and *Fpo* in complex and competitive oral biofilms [30]. Additionally, among the most significantly enriched clusters in *Fpo* was CrcB, a fluoride exporter present in all *Fpo* genomes [29], which may contribute to its dominance in the oral niche [30]. Interestingly, oxidative stress response proteins shared between *Fpo* and *Fvn* further suggest a degree of aerotolerance that may enhance fitness in the gingival crevice [29]. Notably, *Fvn* had only seven specifically enriched gene clusters, four of which have not been fully characterized functionally [30], but it shares some traits favorable to the oral environment, including oxidative stress response proteins and phageassociated elements. Generally, current data indicate that phages may play a role in shaping *Fn* population [29].

### Lipopolysaccharides of F. nucleatum group

Lipopolysaccharide (LPS, endotoxin) is a key component of the outer membrane of Gram-negative bacteria such as *Fn* and in its interaction with the host immune system. Recent studies highlight the diversity of LPS structures across different *Fn* strains, influencing bacterial virulence and immune evasion. Karpathy et al. predicted 25 open reading frames involved in LPS biosynthesis in *Fpo*, interacting with host pattern recognition receptors, particularly the TLR4/MD-2 (myeloid differentiation factor 2) complex, triggering a pro-inflammatory response [13,129].

Structurally, LPS consists of three regions: lipid A, the core oligosaccharide, and the O-antigen. Lipid A, embedded in the outer membrane, is linked to a core region composed of 3‐deoxy‐*d*‐manno‐oct‐2‐ulosonic acid, *l*-glycero-*d*-manno-heptose, and other more common monosaccharides. The O-antigen, with variable repeating units, can consist of up to nine monosaccharides [130–132], with variability observed in the types of sugars incorporated in different *Fn* strains, contributing to strain-specific immune interactions. The characterized LPS structures of multiple *Fn* strains, including *Fnuc* ATCC 23,726 [133], *Fpo* ATCC 10,953 [134], *Fpo* ATCC 12,230 [135] and *Fnuc* ATCC 25,586 [129,136], underscore significant chemical variability and influence the interactions of *Fn* strains with the immune system, thus, affecting their pathogenic potential. While the presence of amino sugars, uronic acids, and amino-acetylating groups is common across strains, there is a marked variability in the carbohydrate composition among strains. The recognition and subsequent signaling cascade are highly dependent on the structure of the lipid A [137,138]. Analysis of the O-antigen and lipid A from *Fan* ATCC 51,191 was characterized and revealed a novel O-antigen repeat unit [131,139,140]. The lipid A structure, similar to *Fnuc* JCM 8532, demonstrated heterogeneity in acyl chains and phosphate content [129,141]. Interestingly, the lipid A from *Burkholderia cenocepacia* exhibits the same acylation pattern as that from *Fan* ATCC 51,191. Although *B. cenocepacia* only expresses tetra‐ and penta‐acylated lipid A, which normally activates human TLR4-MD-2 (myeloid differentiation factor 2) signaling poorly, the lipid A of *B. cenocepacia* strongly activates it despite rather underacylation [142]. Furthermore, the O-antigen of *Fan* ATCC 51191 is characterized by a novel repeating unit that may affect immune cell recognition and cytokine production [131].

Vinogradov et al. investigated the O-chains of different *Fn* strains, revealing short O-chains and molecules with a core that carries one repeating unit of the O-chain [133–136]. The LPS O-chain of *Fnuc* ATCC 23,726 featured a unique 5,7-diamino-3,5,7,9-tetradeoxy-*l*-gluco-non-2-ulosonic acid, which is presumed to have the d-glycero-l-gluco configuration. The O-chain of *Fn* strain MJR 7757 B contained ManNAc4Lac, an analogue of N-acetylmuramic acid, marking its first discovery in nature [134]. Additionally, strain *Fpo* ATCC 10,953 was found to contain sialic acid, supporting earlier evidence of its biosynthesis in the strain [131]. Interestingly, unlike *Fnuc*, *Fpo* lacks the *lic* operon, which typically attaches choline residues to LPS [13,114]. Instead, genes in *Fpo* (FNP_1105–1107) encode enzymes like N-acylneuraminate cytidylyltransferase, N-acetyl neuraminate synthase, and a potential lipooligosaccharide sialyltransferase (FNP_1109), which may allow for sialic acid incorporation into LPS, similar to *Fvn* (note, however, that FNP_1109 is not found in *Fnuc* or *Fvn*) [12,13]. The presence of sialylated LPS may contribute to immune evasion and influence dental plaque formation. Since some LPS molecules of different pathogens exhibit high similarity, it is of great interest whether the host can distinguish between them. The lipid A from *Fnuc* JCM 8532 (originally ATCC 25,586) is similar to compound 506, an *E. coli*-type synthetic lipid A. Notably, *Fnuc* lipid A displayed lower lethal toxicity against d-GalN-sensitized mice. Interestingly, the interaction between lipid A and host cells was influenced by serum components, including fetal bovine serum, sCD14, or a combination of sCD14 and LPS-binding protein, suggesting that sCD14 can detect subtle structural differences between lipid A molecules and influence host cell activation. Structural comparisons of lipid A from various periodontitis-associated bacteria, such as *Aggregatibacter actinomycetemcomitans, Prevotella intermedia*, and *P. gingivalis*, revealed species-specific lipid A compositions that contribute to distinct biological activities. Interestingly, purified lipid A from *P. gingivalis* activated cells via TLR4/MD-2, whereas *P. gingivalis* LPS preparations activated both TLR2 and TLR4/MD-2 due to lipoprotein contamination. Similarly, purified *Fnuc* JCM 8532 lipid A triggered TLR4/MD-2-dependent activation, while its LPS preparation also induced TLR2 signaling. It was suggested that both *Fnuc* JCM 8532 and *P. gingivalis* LPS preparations contain additional molecules that activate TLR2. Contradictory, further experiments demonstrated that *Fnuc* JCM 8532 LPS induced NF-κB activation via TLR4/MD-2 but not TLR2. CD14 appears to enhance the host response of monocytes and macrophages to LPS and the degree of membranous CD14 expression correlates with LPS reactivity. Recent findings indicate that sCD14, but not mCD14, can detect subtle structural differences between lipid A from *Fnuc* JCM 8532 and *E. coli*, leading to distinct host cell activation [143].

Additionally, *Fnuc* ATCC 25,586 LPS increased phospholipase C and D activity, associated with membrane integrity disruption and inflammatory signaling. Elevated prostaglandin E2 levels, linked to bone resorption, correlated with increased phospholipase C activity [144]. Notably, fusobacterial LPS did not induce nitric oxide production in macrophage-like cells, but the possibility of strain-specific differences in nitric oxide response remains open. Unlike in polymorphonuclear neutrophils, *Fn* LPS did not induce apoptosis in macrophage-like cells so far [144–146].

## Sialylation capabilities of *F. nucleatum* group

Sialic acids, a group of nine-carbon sugars derived from neuraminic acid, play a crucial role in cell-to-cell interactions, immune responses, and host-pathogen interactions, particularly on mucosal surfaces [58,147,148]. As a terminal residue on mammalian glycoproteins, it regulates immune cell activation and inflammation [149]. Some bacteria exploit this system through sialylation (glycomimicry), which aids immune evasion [150–154]. Sialylated LPS may protect against serum complement attack [155–157] and suppress immune responses via Siglec receptor (sialic acid-binding immunoglobulin-like receptors) activation [158]. Additionally, mucosal pathogens produce sialidases/neuraminidases to harvest sialic acid from glycoproteins as a nutrient [58,159]. *Fnuc* encodes a sialic acid catabolism operon, inducible by the presence of blood, but does not appear to use sialic acid as a major carbon source [58,148]. The bacterium can synthesize sialic acid *de novo* for surface sialylation, a conserved trait across *Fn* species and *F. periodonticum*. This sialylation is more pronounced in pure cultures than in biofilms, likely due to biofilm-associated sialidases degrading sialylated residues. Sialic acid might also be especially relevant in the context of dental plaque development, where LPS sialylation in fusobacteria could contribute to the ecology of dental biofilms. Additionally, sialic acid in the LPS of *Fn* can be utilized as a carbon source and growth factor by other bacterial species cohabiting dental plaque. For example, previous research has indicated that the utilization of sialic acid from both host and cohabiting bacteria likely helps *Tannerella forsythia* to thrive in the oral environment [160]. Actually, *in silico* analyses of *Fn* have suggested that sialic acid catabolism and cell surface sialylation may occur across various *Fn* species [12,148,161]. Sialic acid catabolism in bacteria typically involves a cluster of genes known as the *nan* genes, which are often organized as an operon. The core enzymes for this process, encoded by the *nanA*, *nanK*, and *nanE* genes, convert Neu5Ac (N-acetylneuraminate) into N-acetylglucosamine 6-phosphate (GlcNAc-6-P). This pathway is associated with various energy-generating and biosynthetic mechanisms. Additionally, *nan* gene loci frequently encode transport systems for sialic acid, which can be ABC transporters, symporters/antiporters, or tripartite ATP-independent periplasmic (TRAP) transporters. The necessary genes for *de novo* sialic acid synthesis are often found in a cluster of *neu* genes, including *neuA*, *neuB*, and *neuC*, which convert UDP-N-acetylglucosamine (UDP-GlcNAc) into N-acetylmannosamine (ManAc) and subsequently to CMP-Neu5Ac, the substrate for sialyltransferases that modify bacterial surface molecules [148]. Although many oral bacteria possess the capability to catabolize sialic acid, the genome sequence data suggests that only a few oral bacteria, such as *Fpo* (in the study of Yoneda et al. the only sequenced *Fn* species), have been shown to encode *neu* gene orthologs [58,161]. Potential *neu* gene operons, which are responsible for sialic acid synthesis, could be found in most sequenced oral fusobacteria genomes, with *Fan* being the sole exception. Currently, it is unclear which genes are responsible for sialic acid synthesis in this species.

When adding defibrinated whole blood at a concentration of 33% to *Fnuc* ATCC 25,586, low levels of free sialic acid in the blood may act as inducers of the *nan* operon. Research suggests that *Fn* strains capable of sialic acid catabolism do not rely on this molecule as a major nutritional source. Actually, high concentrations of sialic acid were found to be toxic for *Fnuc*, possibly due to limited catabolic capacity. The exact advantage of the *nan* operon (in *Fnuc* induced by sialic acid) remains unclear, although it is possible that the high-affinity tripartite ATP-independent periplasmic transporter encoded by this operon may supply exogenous sialic acid for surface sialylation. The other enzymes encoded in the *nan* operon may help prevent toxicity by managing the intracellular accumulation of transported sialic acid. The presence of blood represses various *Fn* pathways but induces *nan* operon expression, aligning with findings that serum sialic acid enhances biofilm-associated bacterial metabolism [58].

Many *Fn* isolates exhibit extensive surface sialylation [58], even in the absence of external sialic acid. *De novo* synthesis may provide a local sialic acid source for biofilm-associated bacteria, such as *T. forsythia*, which relies on sialic acid for growth [157,160,162]. The interplay between fusobacteria and sialidase-producing species within the biofilm likely facilitates metabolic complementation, reinforcing their role in dental plaque ecology. Few bacterial species have been shown to synthesize sialic acid *de novo*, making fusobacteria particularly important for supporting the long-term persistence of mucosal communities.

## Outer membrane vesicles and their role in virulence of *F. nucleatum* group

Outer membrane vesicles (OMVs) secreted by *Fn* contain proteins from various VF families, facilitating protein dispersal and contributing to bacterial survival and immune interactions. These vesicles serve as a mechanism for delivering LPS and other immunomodulatory molecules being capable of activating TLRs on epithelial or immune cells, leading to NF-κB pathway activation and subsequent release of proinflammatory cytokines [79].

OMVs from *Fan* (strain 7_1, EAVG_002) range in size from 40 to 110 nm. Proteomic profiling identified 157 and 190 proteins across two OMV batches, with 98 overlapping. Notably, five of the top 30 inner membrane proteins were also present in the OMVs, including three autotransporters. Cytoplasmic proteins were nearly absent [79]. Actually, many of them were identified as secreted proteins with signal peptides, half of which were likely lipoproteins. Among these, 16 were located in the outer membrane fraction, including extracellular proteins commonly found in OMVs. The presence of inner membrane proteins suggested a double-membrane structure composed of both outer and inner membrane layers. Additionally, five cytosolic proteins appeared to be processed via a non-classical secretion pathway, as they lacked predicted signal peptides. Six autotransporters constituted 31–51% of the total OMV protein load, four of which displayed high similarity, including domains of AidA, a virulence factor in *E. coli* associated with adhesion, autoaggregation, biofilm formation, and invasion [79,163]. Furthermore, two FadA proteins were incorporated into OMVs. The enrichment of autotransporters in OMVs implies a key role in tissue colonization and invasion. Additionally, a protein containing a MORN2 domain, potentially involved in adhesion, was identified. Other surface proteins included YadA-like domains, known virulence factors contributing to adhesion and immune evasion [79]. Notably, OMVs from *Fpo* larger than 50 kDa induced the secretion of proinflammatory cytokines interleukin-8 and tumor necrosis factor in colonic epithelial cells, an effect absent in OMVs smaller than 50 kDa. Inhibition of TLR4 reduced these inflammatory responses, implicating the TLR4 pathway and its downstream effectors p-ERK, p-CREB, and NF-κB. These findings were confirmed in human colonoid monolayers [164].

Furthermore, *Fn* OMVs have recently been shown to carry bioactive nucleic acids, including virulence-associated DNA, which was internalized by intestinal epithelia cells and thus supported the pathogenesis of ulcerative colitis [165]. OMVs can transport diverse RNA species – including mRNA, tRNA, rRNA, sRNA, and sncRNA – which may influence host immune responses and treatment resistance [166].

## Invasive potential of *F. nucleatum* group

**Figure 4.**
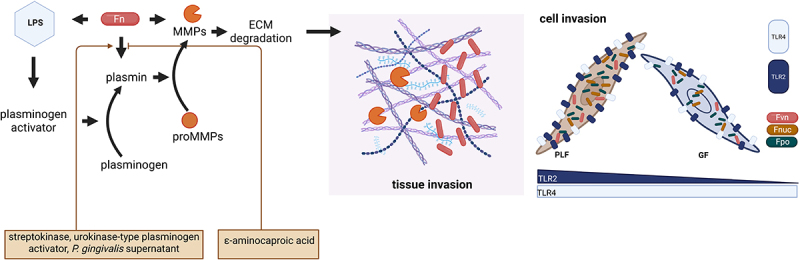
Invasive mechanisms of *Fn*: this schematic summarizes the described invasive potential and virulence mechanisms of *Fn*. Among tested subspecies *Fpo* showed the highest intracellular bacterial mass and *Fvn* the lowest in gingival (GF) and periodontal ligament fibroblasts (PLF). Invasion is strain- and host cell-dependent, potentially mediated via surface ligand – TLR2 interactions and cytoskeletal rearrangement (“zipping” mechanism). *Fn*, particularly *Fnuc*, binds and activates plasminogen on its surface promoting extracellular matrix (ECM) degradation via activation of matrix metalloproteinases (MMPs). *Fnuc* also binds recombinant MMP-9 and pro-MMP-9, enhancing tissue penetration. These mechanisms contribute to *Fn* survival, immune evasion, and pathogenicity in periodontal and mucosal tissues.

*Fn* exhibits invasive capabilities, penetrating host cells and modulating the immune response to promote its survival and dissemination. A recent study demonstrated that different strains of *Fn*, including those from species *Fnuc, Fpo*, and *Fvn*, can invade human gingival fibroblasts (GFs) and periodontal ligament fibroblasts (PLFs), with *Fpo* showing the greatest and *Fvn* the smallest bacterial mass and PLFs generally exhibiting a higher bacterial load than GFs (Figure 4) [167]. Also the invasion in colonic cells was demonstrated [63]. Furthermore, it was reported that *Fnuc* and *Fpo* invaded KB cells, a human oral mucosal epithelial cell line, in nearly equal numbers, suggesting potential intracellular replication and translocation to the extracellular space [38,168]. This suggests that invasion is influenced by both, bacterial strain and host cell type, potentially due to variations in surface ligand expression involved in bacterial recognition and uptake. Intracellular recovery of *Fn* from lysates further implies its capacity for intracellular replication, as previously noted in epithelial cells [167]. Previous research indicated that *Fn* LPS interact with TLRs, contributing to bacterial internalization [169–171]. While TLR4 levels were similar in GFs and PLFs, TLR2 was more abundant on PLFs, aligning with evidence that Gram-negative periodontal bacteria predominantly stimulate TLR2. This differential receptor expression may account for variations in *Fn* uptake between fibroblast types. A “zipping” mechanism was proposed [38] enabling *Fn* to enter human gingival epithelial cells and human oral mucosal cells. This mechanism is supposed to involve localized cytoskeletal rearrangements at the site of bacterial uptake, with bacterial protein synthesis playing a crucial role in the internalization process, similar to observations in oral keratinocytes [167].

This figure was created in BioRender.

This figure was created in BioRender.

It was shown that *Fn* can bind plasminogen and activates it into plasmin, which in turn activates matrix metalloproteinases (MMPs) and degrades extracellular matrix proteins [172]. Plasmin is involved in various physiological and pathological processes, including ovulation, embryogenesis, tumor cell invasion, and tissue destruction at inflammatory sites such as those in periodontitis [172,173]. On the bacterial surface, plasmin degraded fibronectin and tissue inhibitor of metalloproteinase-1 (TIMP-1), promoting tissue invasion [172].

*Fnuc* showed the strongest plasminogen-binding activity among the tested species *(Fnuc, Fvn, Fpo)*. Plasmin-coated *Fnuc* may evade immune responses and enhance virulence. While LPS from *Fnuc* showed no plasminogen-binding, it is possible that plasmin-coated *Fnuc* activates pro-MMPs, particularly pro-MMP-2 and pro-MMP-3, contributing to elevated MMP activity in periodontal lesions [172,174,175]. The degradation of TIMP-1 by plasmin may further increase net MMP activity. Additionally, *Fnuc* ATCC 25,586 LPS was shown to induce the production of a 50 kDa plasminogen activator, MMP-2, and MMP-3, as well as TIMP-1 (but not TIMP-2) in gingival fibroblasts [176]. *Fnuc* ATCC 25,586 LPS interact with CD14 and TLR4 on macrophage-like cells, inducing pro-inflammatory cytokines (IL-1β, IL-6, TNF-α, IL-8) and increasing MMP-9 secretion [144]. *Fnuc* ATCC 25,586 also bound recombinant MMP-9 [177], enhancing their ability to invade tissues. Gendron et al. found that *Fnuc* can acquire and activate cell-associated human pro-MMP-9, increasing its tissue-invasive potential [144,177]. Thus, MMP-9-coated *Fnuc* penetrated a reconstituted basement membrane more effectively [177]. Among tested species – which were *Fnuc* ATCC 25,586, *Fvn* ATCC 49,256, and *Fpo* ATCC 10,953 - *Fnuc* exhibited the highest affinity for pro-MMP-9. This interaction does not seem to involve the previously described plasminogen receptor [172], nor LPS, as shown by experiments using a lipid A-binding inhibitor. MMP-9, which has a fibronectin-like sequence and a collagen-like domain, is known to bind to fibronectin and basement membrane components. This supports the idea that *Fnuc*, known to attach to fibronectin and basement-like matrices [178], might interact with pro-MMP-9 through similar mechanisms. In contrast, *Fnuc* does not bind pro-MMP-8, which lacks such domains. MMP-9 activity on the bacterial surface may contribute to extracellular matrix degradation and cytokine regulation, particularly via activation of IL-1β, since MMP-9 is known to process interleukin-1β from its precursor to its active form [177].

## Immune evasion of *F. nucleatum* group

**Figure 5.**
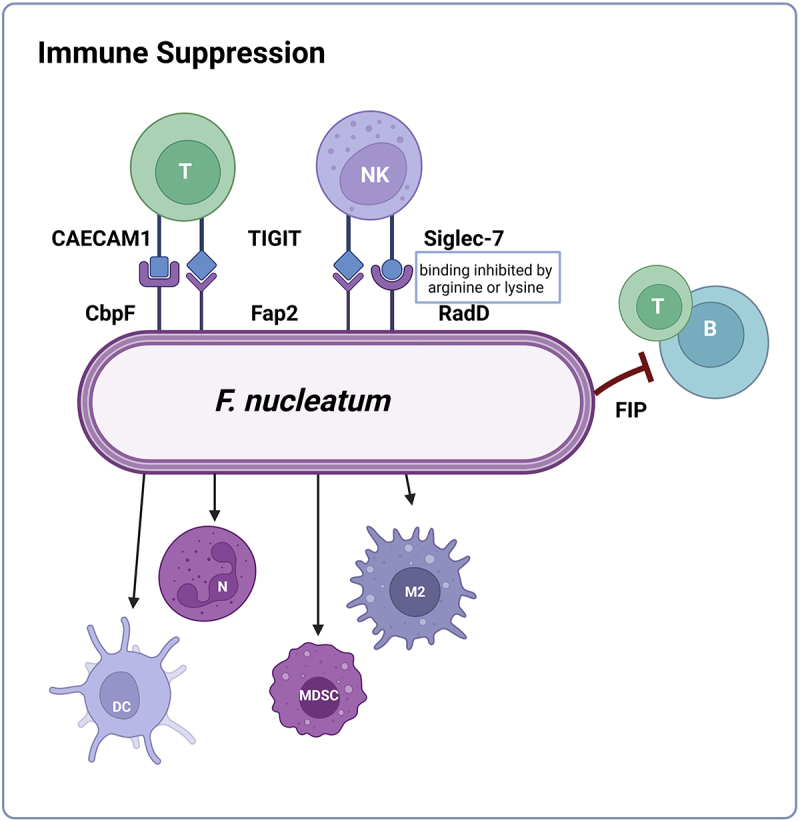
Immune evasion mechanisms of *Fn*: this schematic illustrates the multifaceted immune evasion strategies employed by *Fn* subspecies within the tumor microenvironment and systemic tissues. *Fn* recruits immunosuppressive cells – including tumor-associated macrophages, myeloid-derived suppressor cells (MDSCs), dendritic cells (DCs), and tumor-associated neutrophils (N) – and inhibits T-cell responses of tumor infiltrating lymphocytes, contributing to a pro-tumor inflammatory milieu. A key mechanism involves the engagement of the inhibitory receptor Siglec-7, primarily on NK cells. *Fn* exploits molecular mimicry and RadD (*Fnuc* ATCC 23,726) to bind Siglec-7. In *Fpo* strains lacking RadD it potentially binds Siglec-7 via sialylated glycans. This interaction suppresses NK cytotoxicity, promotes M2 macrophage polarization, and increases IL-10, IL-8, and PD-L1 expression while decreasing CD86, contributing to immune suppression and tumor progression. Strain-specific variations affect binding affinites and immunosuppressive potential. *Fnuc* strains harbor both *radD* and *fap2*, enabling broader receptor targeting, including TIGIT and CEACAM1 on T and NK cells. Additional virulence factors include the fusobacterial immunosuppressive protein (FIP), which inhibits B- and T-cell proliferation, and fad-I, which induces β-defensin 2 via TLR signaling. *Fn* also resists antimicrobial peptides such as defensins through biofilm formation, protease secretion, and activation of resistance pathways.

The ability of *Fn* to modulate host immune responses is a key factor in its virulence and persistence within the host. A notable strategy employed by *Fn* is immune evasion, which allows it to circumvent the host’s immune defenses, promoting bacterial survival and facilitating disease progression. *Fn*‘s role in tumorigenesis and immune evasion involves a complex interplay of interactions with various immune cells and molecules, ultimately leading to an immunosuppressive environment conducive to tumor growth. *Fn* recruits tumor-infiltrating immune cells, including tumor-associated macrophages, myeloid-derived suppressor cells, dendritic cells, and tumor-associated neutrophils [179–181], while simultaneously inhibiting human T-cell responses (Figure 5) [182,183]. This creates a pro-inflammatory profile within the tumor microenvironment, linked to increased NFκB-activation [179]. *Fn* strains exhibit genetic variability, which influences their capacity to evade immune responses.

This figure was created in BioRender.

A key mechanism underlying this immune evasion involves interactions with sialic acid-binding immunoglobulin-like lectins (Siglecs)-transmembrane receptors with immunoregulatory roles. Siglecs are transmembrane proteins characterized by an extracellular V-set immunoglobulin-like domain resembling the antibody variable domain, which contains the carbohydrate recognition domain with specifities to certain sialylated structures [184], and one or more C2-set immunoglobulin-like domains. Most intracellular domains of Siglecs contain immunoreceptor tyrosine-based inhibitory motifs [158]. Siglecs can interact with ligands on the same cell (cis) or on external glycans (trans) [184]. Several bacterial species, including *Fn*, exploit molecular mimicry by displaying sialylated structures to engage Siglecs, thereby evading immune detection [152,185–187]. Each Siglec exhibits specific preferences for sialosides with distinct glycan motifs, contributing to a broad and overlapping ligand specificity [179].

*Fn* specifically binds Siglec-7, an inhibitory receptor predominantly found on natural killer (NK) cells but also expressed on macrophages and dendritic cells. Notably, colonic lamina propria monocytes and macrophages being the major Siglec-7-positive populations [179,188] and Siglec-7 ligands are overexpressed in various human malignancies, leading to the attenuation of NK cell antitumor immunity. Siglec-7 engagement suppresses NK cell cytotoxicity, thereby promoting tumor immune evasion. Ligands for Siglec-7 are overexpressed in various malignancies, correlating with diminished NK cell activity [188,189]. Recent studies confirmed that *Fnuc* ATCC 23,726 binds Siglec-7 through its adhesin RadD, which inhibits NK-mediated tumor cell killing independently of IgA interaction. This interaction depends on arginine residue R124 in Siglec-7 and is inhibited by arginine or lysine and seems to be independent of its interaction with IgA [48,188]. While *Fnuc* exhibits strong RadD-dependent binding to Siglec-7, other species like *Fpo* show little interaction, due to the absence of RadD in some strains like *Fpo* 12,230 [188]. *Fnuc* strains commonly contain both genes, *radD* and *fap2*, *Fan* strains often carry only one [17]. Moreover, strain polymorphisms affect RadD’s binding capabilities. For instance, strain *Fpo* 10,953 lacks RadD but still shows residual Siglec-7 binding, suggesting sialylated glycans may mediate this interaction independently [188,190]. Moreover, RadD of strains *Fnuc* 23,726 and *Fpo* 10,953 binds both Siglec-7 and human serum IgA at likely distinct sites [188,191]. Notably, in a murine preterm birth model using *Fnuc* 23,726, a RadD-deficient mutant showed increased virulence – leading to increased preterm birth rates and elevated bacterial burden in maternal-fetal compartments – potentially due to the absence of Siglec-7 homologs in mice [188,192].

In addition to RadD, *Fan* ATCC 51,191 binds Siglec-7 via its LPS and OMVs, inducing a tumor-promoting phenotype in monocyte-derived dendritic cells and macrophages. This includes upregulation of IL-10, IL-8, and PD-L1, alongside downregulation of CD86, promoting M2 macrophage polarization [113,179,181,193]. Moreover, stimulation with *Fpo* ATCC 10,953, and *Fnuc* ATCC 25,586 resulted in a M2 acquired phenotype in macrophage-like cell lines [181,194]. Both Siglec-7 and Siglec-9 contribute to tumor-associated macrophage polarization and PD-L1 induction [195].

Interestingly, LPS from *Fan* ATCC 51,191 (enriched in CRC tissues [196]) feature unique structural elements, such as a novel O-antigen repeat and bis-phosphorylated hexa-acylated lipid A, likely enhancing Siglec-7 binding. Moreover, O-polysaccharides extracted from this strain were recognized by Siglec-7, revealing new ligand epitopes beyond nonulosonic acids (neuraminic and fusaminic acids). Siglec-7 also appears to synergize with TLR-4, another key receptor recognizing LPS, thus further promoting *Fn*-driven CRC progression and chemoresistance [181,197–199]. Murine Siglec-E, a homolog of Siglec-7, does not recognize *Fn* species, underscoring species-specific immune interactions [200]. Clinically, elevated Siglec-7 expression in CRC tissues correlates with poor immunotherapy outcomes [201], further positioning Siglec-7 as a potential therapeutic target. *Fn* also targets other immune checkpoints: Fap2 binds TIGIT, and CbpF binds CEACAM1 [86,202,203]. While TIGIT is expressed on both T and NK cells and CEACAM1 is found predominantly on activated T cells, Siglec-7 is mainly present on NK cells [204–206]. By targeting multiple inhibitory receptors on different immune cell subsets, *Fn* amplifies immune suppression and sustains an immunosuppressive environment making it a critical target for immune evasion [86,188,202,203].

Strain-specific virulence is also shaped by other proteins such as Fad-I, which induces human β-defensin 2 (hBD-2) through TLR signaling. Stronger immune modulation was noted in ATCC-strains *Fnuc* 23,726 and 25,586 compared to *Fpo* 10,953 [36]. Gerardo et al. identified another VF: the fusobacterial immunosuppressive protein (FIP), particularly its subunit FipA. Highly conserved across *Fn* strains and cloned from *Fpo* (ATCC 10,953) and *Fnuc* (ATCC 23,726), FIP inhibits B- and T-cell proliferation by arresting cells in G0/G1, although its metabolic role remains unclear [207–209]. Furthermore, several bacteria, including *Fn*, counter effects of antimicrobial peptides through protease production, AMP-binding proteins, biofilm formation, and two-component regulatory systems [210].

Additionally, the ability to interfere with neutrophil activity differs among (sub-)species [18]. Specifically, *Fnuc* and *Fpo* are capable of fully suppressing superoxide generation, whereas *Fvn* exerts only a partial inhibitory effect. Moreover, *Fpo* showed a greater propensity to trigger neutrophil apoptosis than either *Fnuc* or *Fvn* [18,211].

## Biofilm-forming capacity of *F. nucleatum* group

*Fn* is a keystone species in oral biofilms, acting as a physical and metabolic bridge between early and late colonizers. Its different species exhibit unique binding properties, contributing to the microbial ecology of various niches. Biofilm architecture, thickness, and stability varies between *Fn* species [212,213].

In coaggregation with *Streptococcus mutans*, the surface adhesin SpaP in *S. mutans* binds specifically to *Fpo*, but not to other *Fn* species [214]. SpaP, anchored by SrtA, interacts exclusively with RadD, indicating a species-specific interaction, although RadD homologs are present in other fusobacterial species. In contrast, *Fnuc* interacts with *S. mutans* through RadD via a different, likely non-SrtA-dependent, partner. Sequence analysis suggests that SpaP binding involves RadD’s variable regions (amino acids 285–625 or 1716–1811), unique to *Fpo*. RadD also mediates attachment of *Fnuc* to *S. sanguinis* and *S. gordonii* [48].

Interestingly, *Fusobacterium* species are frequently co-colonizers in *Clostridioides difficile* infection (CDI), contributing to gut microbiota disruption [215]. *C. difficile* coaggregates in vitro with multiple *Fn* species (*Fnuc, Fpo, Fan*), a process mediated by *C. difficile* flagella binding to RadD. This interaction enhances *C. difficile* biofilm formation and extracellular polysaccharide production, suggesting *Fn* as a disease-modifying factor. *Fn* species adhered to purified MUC2 and MUC2-producing HT29-MTX cells, confirming their ability to colonize the intestinal mucus layer. While only a subset of CDI patients harbored detectable fusobacteria, its abundance was higher in patients with diarrhea but negative for CDI, implying a broader role in dysbiosis [215]. Coaggregation assays confirmed RadD as the primary mediator, with *Fan* exhibiting the weakest interaction due to lower auto-aggregation capacity. RadD deletion significantly impaired coaggregation, while Fap2 deletion had a modest effect [215].

Additionally, all most prominent oral *Treponema* species (*T. denticola*, *T. socranskii*, *T. vincentii*, *T. pectinovorum*) coaggregated with at least one *Fusobacterium* strain, including *Fnuc, Fpo, Fvn*, and *F. periodonticum*. Coaggregation was protein-dependent: heat-inactivated fusobacteria lost the ability to bind, while treponemes remained unaffected. Co-aggregation between *Fn* species and *Treponema* spp. was highly specific, with no coaggregation observed among different *Treponema* species themselves. The coaggregation specificity varied among *Fn* species. For instance, *Fvn* ATCC 49,256 resisted proteinase K, while *Fpo* ATCC 10,953 showed partial resistance. Other fusobacteria tested were completely inactivated by proteinase K treatment. Pronase partially inhibited binding (on *Fnuc* PK1594), whereas trypsin did not, highlighting the role of protein-based adhesins over carbohydrate-mediated interactions. Some *Fn* species also coaggregated with each other and with *F. periodonticum*, forming strain-dependent interaction profiles [216].

*Helicobacter pylori* also exhibited strong coaggregation with all four *Fn* species from human dental plaque (*Fnuc* PK 1594, *Fpo* ATCC 10,953, *Fvn* ATCC 49,256, *Fvn* NCTC 11,326), but not with other tested strains [217]. Again, heat-inactivated *Fn* lost this ability, while *H. pylori* remained unaffected, suggesting *Fn* expresses the adhesin and *H. pylori* carries the receptor. The interaction appears to involve fusobacterial protein adhesins and *H. pylori* carbohydrate receptors. This supports the idea that the oral cavity, particularly saliva, could play an important role in the infectious cycle of *H. pylori*, although this topic is discussed controversially [218–220]. Nevertheless, many interactions are based on in vitro systems and require further in vivo validation.

*Fnuc* PK1594 also coaggregated with late-colonizing Gram-negative oral bacteria like *P. gingivalis* in a lactose-inhibitable manner [221]. Moreover, the abundance of *Corynebacterium matruchotii* was found to correlate with *Fpo*, highlighting intricate microbial interactions within oral biofilms [222].

Interestingly, Wu et al. identified nine distinct genetic lineages of *Fusobacterium* (L1–L9) and demonstrated that these lineages differ markedly in their microbial co-aggregation and co-occurrence patterns. Using metagenomic network analyses, they showed that the CRC-associated lineage L1—comprising *Fn*, *F. periodonticum*, *F. pseudoperiodonticum*, *F. hwasookii*, and closely related species – preferentially co-occurred with biofilm-associated taxa, including *Streptococcus*, *Campylobacter*, and *Gemella* species. Notably, *Streptococcus* and *Gemella* are core members of the oral microbiota [32,223,224], and their well-established ability to coaggregate with *Fn* suggests that L1 participates in biofilm-like microbial assemblies. For a detailed description of the network analysis and lineage-specific interactions, see Wu et al. [32].

In summary, the intricate web of interspecies interactions highlights the complexity of these relationships in various ecological niches as well as the importance of further investiagtions regarding (sub-)species specific lineages and disease-associated coaggregation patterns.

## Lyases of *F. nucleatum* group

*Fn* is among the most potent hydrogen sulfide (H_2_S) producers in the human oral cavity [225–227]. H_2_S is implicated in various biological effects, including hemoglobin modification in erythrocytes [228,229], endotoxin-induced inflammation [230], and apoptosis of both aortic smooth muscle cells and human gingival fibroblasts [231,232]. These effects are particularly relevant to periodontal disease, where H_2_S concentrations in periodontal pockets are markedly elevated and with multiple H_2_S-producing enzymes identified across studies [233,234].

*Fnuc* actively transports small peptides, efficiently processing sulfur-containing compounds. Notably, among periodontal bacteria peptides were being metabolized more efficiently than free amino acids. *Fnuc* can transport L-cysteinylglycine but not glutathione, highlighting substrate specificity [235,236].

Several key H_2_S-producing enzymes have been identified in *Fnuc* ATCC 25,586. Suwabe et al. characterized Fn0625, Fn1055, Fn1220, and Fn1419, ranking their enzymatic productivity as follows: Fn1220 > Fn1055 > Fn1419 > Fn0625 with Fn1220 alone accounting for 87.6% of the total H_2_S production [237]. Fn1220 catalyzes a β-replacement reaction condensing two L-cysteine molecules to yield H_2_S and L-lanthionine [235, 240]. Fn1055, a pyridoxal 5’-phosphate (PLP)-dependent cysteine hydroxyl lyase, converts L-cysteine to L-serine and H_2_S via a β-replacement mechanism [233]. Key catalytic residues include Asp232 (critical for L-cysteine specificity), Ser74, Thr73, and Gln147. Because *Fn* lacks L-serine biosynthesis genes, Fn1055 likely plays a regulatory role in L-serine availability [225].

Fn0625 catalyzes α,β-elimination of L-cysteine to yield H_2_S, pyruvate, and ammonia [238]. Fn1419, found in all (sub-)species, has dual activity: it catalyzes α,γ-elimination of L-methionine (producing methyl mercaptan, α-ketobutyrate, and ammonia) and α,β-elimination of L-cysteine producing H_2_S, pyruvate, and ammonia [239,240]. However, the *l*-cysteine degradation activity of *l*-methionine γ-lyase from *Fpo* ATCC 10,953 was lower than its *l*-methionine degradation activity, suggesting that *l*-cysteine is not a preferred substrate for this enzyme. Despite its lower kinetic efficiency, Fn1419 shows the highest activity in crude extracts (SDS-PAGE) [240]. Beyond L-cysteine, at least *Fnuc* ATCC 25,586 can also generate H_2_S from other substrates such as L-homocysteine and potentially glutathione [236,240]. Enzyme expression levels are likely influenced by environmental and growth conditions [237].

*Fn* also plays a role in metabolizing cysteine conjugates – such as propyl-, 2-heptyl-, and 3-hexanol-cysteine – into odorant thiols, implicating its carbon-sulfur (C-S) lyases in oral malodor [241]. Microbial PLP-dependent C-S lyases likely contribute to this transformation, with PLP enhancing and L-cycloserine inhibiting the process. PatB-like C-S lyases, part of the type-I PLP-dependent aminotransferase family, catalyze C-S bond cleavage via Schiff base formation, leading to thiol, pyruvate, and ammonia production [242–244]. Among these, FnaPatB1 from *Fan* demonstrated superior activity with various cysteine conjugates compared to Fn0625 from *Fnuc*, particularly for methyl- and ethyl-L-cysteine derivatives [238]. Its activity was reduced by up to 30% by quercetin and curcumin, suggesting a possible role in odor mitigation. The presence of this enzyme in *Fan* but not in other (sub-)species raises questions about gene acquisition or loss, potentially via HGT. Genomic analysis revealed that Fn0625-like sequences with high identity ( > 90%) exist in all *Fn* species, pointing to a conserved role possibly related to homocysteine metabolism. Additionally, a second group of more distantly related PatB sequences (≈40% identity) was identified, with at least one member found in each *Fn* species. This suggests a distinct enzymatic function separate from Fn0625-like lyases [242].

*Fn* possesses a diverse arsenal of sulfur-metabolizing enzymes, with H_2_S production driven by substrate specificity, enzymatic diversity, and environmental factors.

## Conclusions

*Fn* exhibits diverse mechanisms that enhance its adaptability, pathogenicity, and persistence in polymicrobial communities. Variations in LPS structure, sialylation, immune evasion, oxidative stress resistance, and biofilm formation highlight its complex interactions with the host. *Fn* species rely on the TVSS, with secretion system genes showing partially *Fn*-species-specific patterns. *Fpo* has the broadest range of variants (TVa – TVe), whereas *Fnuc* and *Fvn* are limited to TVa – Vc. Fap2 is the most conserved TVa adhesin across (sub-)species, whereas RadD varies, being less common in *Fpo* and probably a key-molecule for Siglec-7 binding in *Fnuc*. FadA is unique to oral fusobacteria and conserved in *Fn*. *Fpo*’s genome is large and diverse, likely due to horizontal gene transfer, featuring various ABC transporters and *neu* operons for sialic acid use and immune mimicry, less common in other *Fn* species. Coaggregation differences, such as *Fpo*’s unique interaction with *S. mutans*, further demonstrate *Fn*‘s functional plasticity. These findings emphasize the need for further (sub-)species specific analysis to understand their different roles in health and disease and to develop targeted interventions for *Fn*-associated pathologies.

## Data Availability

Data sharing is not applicable to this article as no new data were created or analyzed in this study.
